# Distinct Local and Global Dynamics of α‑Helices and β‑Sheets in Poly(γ-benzyl‑l‑glutamate) Peptides

**DOI:** 10.1021/acs.biomac.5c02128

**Published:** 2025-12-19

**Authors:** Marianna Spyridakou, Iren G. Stavrakaki, Evangelia Tsagkaraki, Christina Varfi, Robert Graf, Hermis Iatrou, George Floudas

**Affiliations:** 1 Department of Physics, 37796 University of Ioannina, P.O. Box 1186, Ioannina 45110, Greece; 2 Department of Chemistry, 68993 University of Athens, Athens 15771, Greece; 3 28308 Max Planck Institute for Polymer Research, Ackermannweg 10, Mainz 55128, Germany; 4 Institute of Materials Science and Computing, University Research Center of Ioannina (URCI), Ioannina 45110, Greece

## Abstract

A series of poly­(γ-benzyl-l-glutamate) (PBLG) peptides are synthesized, with a broad range of molar masses and different end-groups. By combining static (wide-angle X-ray scattering and ^13^C solid-state nuclear magnetic resonance (NMR)) with dynamic probes (^13^C solid-state NMR, dielectric spectroscopy (DS) as a function of temperature and pressure, and rheology), we could identify distinct local and global dynamics associated with α-helices and β-sheets. The local dynamics reflect segmental relaxations of amorphous segments interrupting the α-helices/β-sheets and at their chain-ends. Two glass temperatures (*T*_g_s) were identified in oligopeptides exhibiting both secondary structures. This is the first report for β-sheet-associated *T*_g_ in completely nonhydrated polypeptides. At longer timescales, the relaxation of the α-helical and β-sheet macrodipoles was also evident in DS. Peptides with different secondary structures have distinct viscoelastic signatures. Overall, polypeptide chain length and end-group chemistry can be employed to engineer α-helices and/or β-sheets, enabling deliberate control over the structural, dynamical, and viscoelastic properties.

## Graphical abstract

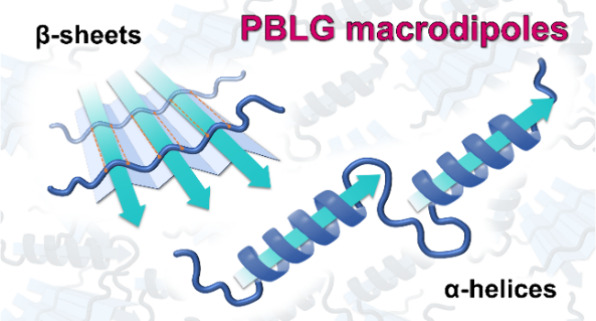

## 1 Introduction

Proteins achieve their remarkable functional diversity due to the specialized roles of their secondary structural motifs, primarily α-helices and β-sheets. α-Helices provide mechanical flexibility for dynamic processes such as oxygen binding and signaling, while β-sheets provide rigidity for resilience in harsh environments and functions such as antigen recognition.^1^ Mixed α/β domains balance these traits, which is crucial for proper function. These characteristics are currently being leveraged in protein engineering to develop biomaterials and therapeutics, highlighting the relationship between the secondary structure and biological function.^2^

Evidently, these structural motifs are dynamic in nature. Protein dynamics depend on temperature, with early studies showing that proteins undergo a liquid-to-glass “transition”, independent of their structure.^3,4^ Debate centered on whether this transition is intrinsic or water-mediated. To this end, several computational^5,6^ and experimental^7–13^ works explored the dynamic arrest in hydrated proteins. The presence of hydration water appears to significantly modulate the peptide molecular mobility, leading to the so-called “slaving” of the peptide dynamics.^7^ However, the exact definition of “slaving” remained somewhat ambiguous. Although similarities in the temperature dependence of protein and solvent relaxations suggested some kind of dynamic coupling, the processes involved often differed in timescale by several orders of magnitude, while the hydration level strongly controlled the extent of the effect. Studies of model polypeptides in their bulk state were very instrumental for this problem as they confirmed the existence of a liquid-to-glass “transition” in the absence of a solvent, e.g., a property intrinsic to the polypeptides.^14–16^

In α-helical polypeptides, the liquid-to-glass “transition” was assigned to defected hydrogen bonds interrupting the α-helical regions. Furthermore, the α-helices themselves were found to be dielectrically active due to their axial dipole moment, giving rise to a characteristic relaxation process known as the “slow helix process”.^14–16^ On the contrary, for β-sheet proteins, such as *Bombyx mori* silk, a high-temperature glass “transition” was proposed,^17^ but evidence suggested that it could originate from bound water.^18^ Hydration and crystallinity complicated the detection of this “transition”, while the strong conductivity at high temperatures obscured the dielectric analysis.^19–21^ Despite recent simulations indicating that β-sheets also possess macrodipoles,^22^ their dynamics remained largely unexplored.

This background raises several scientific questions:

1. Do β-sheets exhibit a liquid-to-glass “transition” analogous to that of α-helices, and if so, what is its molecular origin? Directly comparing the dielectric properties of α-helical and β-sheet polypeptides could elucidate molecular-level similarities and differences between the two secondary structures.
2. What are the characteristic length- and timescales associated with the relaxation of β-sheets? Beyond the contribution of amorphous segments to the dynamics, we aim to investigate the intrinsic relaxation of ordered β-sheets. Recent simulations have indicated that they process a significant dipole moment^22^ and thus have the potential for a distinct dielectric response.
3. How are β-sheets affected by pressure? Studies that consider both temperature and pressure are of interest, as β-sheets operate in diverse environments (e.g., silk’s responsiveness to humidity and amyloid pathology under cellular stress). This could enable better control of β-sheet assembly for applications ranging from tunable biomaterials to the inhibition of amyloidogenesis.
4. Do β-sheets exhibit a distinct viscoelastic signature as compared to α-helices? Understanding their viscoelastic behavior could reveal how β-sheet-rich and α-helix-rich polypeptides align with the stiffness of various biological tissues. In particular, how do their moduli compare to those of classical biomedical materials, such as drug delivery matrices (typically in the kPa range, designed to be soft for biocompatibility and degradation) or plastic surgery implants (designed stiffer, ranging from kPa to GPa, for structural support)? Representative examples include the human femur (∼15 GPa), Achilles tendon (∼1 GPa), retina (∼20 kPa), epithelial tissues (∼1 kPa), or anterior basement membranes (∼4 kPa).^23,24^

To address these questions, we synthesize a series of poly­(γ-benzyl-l-glutamate) (PBLG), a model synthetic polypeptide, with different end-groups and controlled degrees of polymerization. The series include short to intermediate oligopeptides that exhibit both secondary structures as well as polypeptides dominated by α-helical structures. Subsequently, we employ different techniques probing distinct features related to thermodynamics (TM-DSC), structure (X-rays), dynamics (solid-state NMR and dielectric spectroscopy as a function of temperature and pressure (DS)), and viscoelasticity (rheology). The results show two distinct glass temperatures (*T*_g_s) in polypeptides that exhibit both secondary structures. The lower *T*_g_ is attributed to the amorphous segments interrupting α-helices, while the higher *T*_g_ to the amorphous segments interrupting ordered β-sheets. The slower segmental dynamics of the β-sheets indicate significantly more restricted dynamics as compared to helices. The different fragility values and pressure dependences of the α-helices and β-sheets are discussed in terms of the different structural environments imposed by the type of secondary structure. Specifically, the network-like structure of β-sheets imposes stronger constraints on the nearby amorphous segments. Global dynamics of macrodipoles along (α-helices) and perpendicular (β-sheets) to the chains were identified through dielectric spectroscopy. Lastly, we find an elastic response (*G′* > *G″*) across all polypeptides. Through the analysis of the van Gurp–Palmen (vGP) plot, we show that the different secondary structures have a clear viscoelastic signature at the segmental level and a similar fingerprint at the domain level, which reflects the emergence of a supramolecular structure, i.e., a “mesh”, that decreases in size with increasing molar mass.

## 2 Experimental Section

### 2.1 Synthesis and Characterization of the Polypeptides

#### 2.1.1 Synthesis and Characterization of γ-Benzyl-l-glutamate *N*-Carboxy Anhydride (BLG-NCA)

The NCA of γ-benzyl-l-glutamate was synthesized following a previously reported method.^25^ Briefly, γ-benzyl-l-glutamate was suspended in dry ethyl acetate, after which limonene and triphosgene were added. The mixture was heated at 75 °C until the solution turned clear, indicating the formation of the NCA. The solvent was distilled off in the vacuum line, and fresh dry ethyl acetate was distilled in the flask, to dissolve the crude NCA, followed by removal of the solvent by distillation. The resulting γ-benzyl-l-glutamate *N*-carboxy anhydride (BLG-NCA) underwent further purification through two recrystallizations from dry ethyl acetate and *n*-hexane under high vacuum.

To confirm the NCA’s purity, IR spectra of both the precursor H-Glu­(OBzl)–OH and the final product were obtained (Figure S1, Supporting Information). The broad peak between 2500 and 3600 cm^–1^ corresponds to the −N–H stretching vibration of the primary amine, along with −C–H stretches from the carbon skeleton and aromatic ring. The ester carbonyl at 1723 cm^–1^ and aromatic ring vibrations between 600 and 800 cm^–1^ were observed. Peaks at 1621 and 1514 cm^–1^ correspond to the bending of the N–H bond, while the 1580 cm^–1^ peak confirms the dipolar ionic form of the amino acid. In the final product spectrum, the appearance of two peaks at 1867 and 1844 cm^–1^, which correspond to the symmetrical and asymmetric vibration of the NCA carbonyls, respectively, indicates the successful synthesis of *N*-carboxy anhydride. No amide bond peak at 1650 cm^–1^ was observed, excluding the possibility of premature polymerization. Additionally, the purity of γ-benzyl-l-glutamate NCA was confirmed by ^1^H NMR in CDCl_3_ (Figure S2).

#### 2.1.2 Synthesis and Characterization of Poly­(γ-benzyl-l-glutamate) (PBLG)

All polymers were synthesized by splitting the same batch of monomer, excessively purified DMF, as well as dimethylamine (DMA) initiator at the appropriate amounts. The reactions used for the synthesis of PBLG homopolymers are shown in Scheme S1. The polymerizations were performed in a custom-made apparatus equipped with a high-vacuum stopcock for periodic degassing of the solution. The apparatus, containing a magnetic stirring bar, was initially attached to the vacuum line through the ground joint. It was evacuated and flame-dried several times and subsequently was transferred to the glovebox where the calculated quantity of BLG-NCA was added. The apparatus was attached to the vacuum line, and after thorough evacuation of the monomer, the middle fraction of highly purified DMF was distilled into the apparatus. The monomer was dissolved, and the solution of the initiator dimethylamine (DMA) dissolved in pure DMF was added through the rupture of the break-seal of the ampule. The solution was vigorously stirred and periodically pumped to remove the CO_2_ produced from polymerization. The consumption of the monomer was monitored by FT-IR through removal of an aliquot under an argon atmosphere. It was observed that the initial NCA was completely consumed, as the characteristic peaks of the carbonyls of the cyclic NCA and the carboxyl group in the range of 1630–1510 cm^–1^ are absent, while at the same time, the absorption of the ester bond of the side group remains intact. As an example, the amounts used for the synthesis of the homopolymers with a degree of polymerization of 7 (stoichiometric *M*_*w*_ = 1577 g mol^–1^) are the following: 1.7 mL of dissolved DMA in DMF with the concertation of 1.289 × 10^–4^ mol mL^–1^ was added to 0.4034 g of the monomer dissolved in DMF. After completion of the polymerization, the polypeptides with a higher molecular weight were precipitated in diethyl ether and dried under high vacuum, while the polypeptides with a molecular weight under 2000 g mol^–1^ were concentrated and dried under HV.

#### 2.1.3 Fourier Transform Infrared Spectroscopy

(FT-IR) measurements were performed with a PerkinElmer Spectrum One instrument, recorded in KBr pellets at room temperature across a range of 450–4000 cm^–1^.

#### 2.1.4 Proton Nuclear Magnetic Resonance Spectroscopy

^1^H NMR (400 MHz) was conducted on a Bruker 400 spectrometer. The spectra of the polymeric materials as well as the monomers (*N*-carboxy anhydrides (NCAs)) were recorded in CDCl_3_, at ambient temperature using Bruker’s standard pulse programs for proton ^1^H and COSY.

#### 2.1.5 Size-Exclusion Chromatography (SEC)

SEC analysis was performed using two SEC sets. One was composed of a Waters Breeze instrument (Milford, MA, USA) equipped with a 2410 differential refractometer and a Precision PD 2020 two-angle (15° and 90°) light scattering detector (TALLS). The carrier solvent was 0.05% potassium trifluoroacetate (KTFA) solution of HFIP at a flow rate of 1 mL min^–1^ at 40 ^ο^C. A PSS PFG precolumn, a PSS PFG 100 Å, and a PSS PFG 1000 Å column placed in series were employed. The concentration that was utilized was 1 mg mL^–1^, and sonication was performed when needed. A second SEC instrument was used for the analysis of the polypeptides. The system was composed of a Waters 600 HPLC pump, Waters Ultrastyragel columns (HT-2 and HT-4), a Waters 410 differential refractometer, and a Precision PD 2020 two-angle (15° and 90°) light scattering detector (TALLS) operating at 60 °C. The carrier solvent used was a solution of 0.1 M LiBr in DMF with a flow rate of 1 mL min^–1^. The concentration measured was 6 mg mL^–1^, and sonication was performed when needed.

### 2.2 Methods of Physical Property Characterization

#### 2.2.1 Wide-Angle X-ray Scattering

Wide-angle X-ray scattering (WAXS) measurements were performed with a D8 Advance Bruker diffractometer, Cu Kα (40 kV, 40 mA) radiation, equipped with a secondary beam graphite monochromator (λ = 1.54184 nm). The system employed a Bragg–Brentano geometry in a θ–θ configuration. Patterns were obtained over the range of 2θ from 2° to 45° in steps of 0.01°, and the rate was 2 s per step for all samples. The recorded intensity distributions are presented as a function of the modulus of the scattering vector. Scattering curves were taken at a temperature of 303 K.

#### 2.2.2 Solid-State NMR

^13^C CP MAS NMR spectra were recorded with a Bruker Avance III NMR console operating at a 500.20 MHz ^1^H Larmor frequency at an 11.7 T Oxford Instruments wide-bore NMR magnet using a commercial double-resonance CP MAS probe supporting zirconia MAS NMR rotors with a 2.5 mm outer diameter at a 25 kHz magic-angle spinning frequency. The rf power was adjusted on both channels, ^1^H and ^13^C, to a 100 kHz rf nutation frequency. A 90–100% ramped CP contact pulse was used on the ^1^H channel, in order to account for possible rf instability and off-resonance conditions, and the duration of the CP contact time was 1 ms. High-power swept-frequency TPPM decoupling^26^ with a 100 kHz rf nutation frequency was applied on the ^1^H channel during acquisition. The sample temperatures under fast MAS spinning conditions were corrected for frictional heating in the air bearings using the temperature-dependent chemical shift of lead nitrate.^27^ The conformation-dependent NMR signals of the polypeptides were assigned according to Shoji et al.^28^ The quantitative analysis of the NMR spectra was performed by spectral fitting using DMfit software.^29^ The molecular dynamics investigations were performed by recording ^13^C–^1^H REREDOR spinning sideband patterns^30,31^ at an 80 μs recoupling time and a 25 kHz magic-angle spinning (MAS) on a Bruker Avance 500 spectrometer using Bruker double-resonance probe supporting rotors of 2.5 mm outer diameter. Temperatures were corrected for frictional heating effects arising from the fast rotor spinning.^32^

#### 2.2.3 Temperature-Modulated Differential Scanning Calorimetry (DSC)

A Q2000 (TA Instruments) was used for the thermal analysis. The instrument was calibrated (including baseline calibration) for best performance in the specific temperature range and heating/cooling rate using a sapphire standard. Samples were sealed in an aluminum pan, and an empty pan was used as the reference. A low-frequency sinusoidal signal was applied to the standard DSC profile as *T* = *T*_0_ + β*t* + *A*_T_ sin­(ω*t*), where β is the rate, *A*_T_ is the amplitude (typically 1 K), and ω is the angular frequency. The rate/period pair was employed for each measurement according to $\beta =\frac{\Delta T_{g}}{cP}60\;s/\min$ . Here, ΔΤ_g_ is the breadth of *T*_g_, *c* is the number of cycles across the *T*_g_ width, and *P* is the oscillation period. The period/rate pairs used were as follows: 20 s/10 K min^–1^, 40 s/5 K min^–1^, and 60 s/3.3 K min^–1^. The temperature range for the TM-DSC measurements was from 230 to 350 K.

#### 2.2.4 Dielectric Spectroscopy

Dielectric spectroscopy (DS) measurements as a function of temperature (*T*) and pressure (*P*) were performed with a Novocontrol Alpha frequency analyzer. The *T*-dependent measurements at ambient pressure were performed within the range from 223 to 423 K in steps of 5 K for frequencies in the range from 10^–2^ to 10^7^ Hz. The sample cell consisted of two electrodes, 20 mm in diameter and 50 μm in thickness, maintained by Teflon spacers. Samples were prepared at 353 K under vacuum by pressing the electrodes to the spacer thickness where necessary. The *P*-dependent protocol involved measurements from 323 to 503 K in a Novocontrol pressure cell. The pressure setup consisted of a temperature-controlled cell, a hydraulic closing press with an air pump, and an air pump for hydrostatic test pressure. The preparation of the sample capacitor began with the sample pressed under vacuum between 20 mm-diameter electrodes and a thickness of 50 μm maintained with Teflon spacers. Subsequently, the capacitor was wrapped with Teflon tape and arranged inside a Teflon ring in order to prevent the flow of silicon oil into the sample. Silicone oil (DOW CORNING 550 Fluid) is the liquid that uniformly transmits pressure to the sample. The isothermal measurements were made with temperature stability better than 0.1 K and pressure stability better than 2 MPa. The complex dielectric permittivity ε* = ε′ – *i*ε″, where ε′ is the real and ε″ is the imaginary part, was obtained as a function of frequency, ω, temperature, *T*, and pressure, *P*, i.e., ε*­(Τ, *P*, ω).^33,34^ The analysis of the DS curves was based on the empirical equation of Havriliak and Negami (HN)

$$\varepsilon^{*}\left(\omega ,Τ,P\right)=\varepsilon_{\infty }+\sum_{k}\frac{\Delta \varepsilon_{k}\left(Τ,P\right)}{{\left[1+{\left(i\times \omega \times \tau_{\mathrm{HN},k}\left(Τ,P\right)\right)}^{m_{k}}\right]}^{n_{k}}}+\frac{\sigma_{0}\left(Τ,P\right)}{i\varepsilon_{0}\omega }$$ 1

where Δε_*k*_ is the dielectric strength, τ_HN,*k*_ is the H–N characteristic relaxation time, *m*_*k*_ and *n*_*k*_ (with limits 0.2 < *m*_*k*_ and *mn*_*k*_ ≤ 1) describe, respectively, the symmetrical and asymmetrical broadening of the distribution of relaxation times, and the index *k* indicates the process under investigation. At lower frequencies, the dielectric loss sharply rises due to the conductivity contribution as σ_0_/ε_0_ω, where σ_0_ is the dc conductivity and ε_0_ (= 8.854 × 10^–12^ F m^–1^) is the permittivity of free space. From τ_ΗΝ,*k*_, the relaxation times at maximum loss, τ_max_, were obtained analytically from the HN equation as follows

$$\tau_{\max,k}=1/2\pi f_{\max,k}=\tau_{\mathrm{HN},k}{\left[\sin\left(\frac{\pi m_{k}n_{k}}{2\left(1+n_{k}\right)}\right)/\sin\left(\frac{\pi m_{k}}{2\left(1+n_{k}\right)}\right)\right]}^{1/m_{k}}$$ 2

These relaxation times correspond to the relaxation times of the segmental processes. Except for the measured ε″, the derivative of the real part of the dielectric permittivity, ε′, $\left(\frac{d\varepsilon^{\prime }}{\mathrm{dln}\;\omega }\approx -\left(2/\pi \right)\varepsilon^{\prime \prime }\right)$ applicable for broad peaks was used in the analysis of the dynamic behavior.^35^

#### 2.2.5 Rheology

A TA Instruments, AR-G2, with a magnetic bearing that allows for nanotorque control was used to record the viscoelastic properties of PBLG as a function of molar mass. Measurements were made with the environmental test chamber (ETC) as a function of temperature. Samples were prepared on the lower plate of the 8 mm-diameter parallel plate geometry or as pellets by compression molding. Temperature control was achieved within 0.1 K with a nitrogen convection oven. The linear and nonlinear viscoelastic regions were determined by the strain amplitude dependence of the complex shear modulus |G*| at ω = 10 rad s^–1^. The storage (*G′*) and loss (*G″*) shear moduli were measured as a function of frequency, ω, within the range of 10^–1^ to 10^2^ rad s^–1^ at several temperatures from 293 to 493 K. Master curves were constructed by using the time–temperature superposition principle (*tT*s). The *tT*s principle allows the frequency dependence of G* at any temperature to be determined from a master curve constructed at a reference temperature, *T*_ref_, as G*­(ω, *T*) = *b*_T_ G*­(α_T_ω; *T*_ref_). The shift factors, α_Τ_, were fitted according to the Williams–Landel–Ferry (WLF) equation as $\alpha_{Τ}=\frac{-C_{1}^{\mathrm{ref}}\left(T-T_{\mathrm{ref}}\right)}{C_{2}^{\mathrm{ref}}+\left(T-T_{\mathrm{ref}}\right)}$ , where $C_{1}^{\mathrm{ref}}$ and $C_{2}^{\mathrm{ref}}$ are empirical parameters at *T*_ref_.

#### 2.2.6 Thermal History

First, all samples were annealed at 160 °C for 4 days prior to the rheological, dielectric, and DSC measurements to remove any residual solvent and to erase any thermal history. Following this annealing step, the powder samples were used directly without any additional solvent-casting or molding steps in DSC and XRD. For the DS and rheology experiments, we followed two sample preparation protocols: In the first case, samples were molded by heating on the lower electrode plate used in DS or on the lower plate of the rheometer. In the second case, samples were prepared by compression molding and subsequently inserted in the DS capacitor and rheometer plates. No evidence of history-dependent effects was observed in DS and rheology.

## 3 Results and Discussion

### 3.1 Synthesis and Characterization of the Polypeptides

Poly­(γ-benzyl-l-glutamate) (PBLG) is a model synthetic polypeptide that adopts both secondary structures, α-helices and β-sheets, with the relative fraction depending on molecular parameters such as the degree of polymerization and the dispersity.^14–16^ These structures, stabilized respectively by intramolecular and intermolecular hydrogen bonds in α-helices and β-sheets, have an effect on the self-assembly, the dynamics, and the viscoelastic response. In this study, two series of PBLG samples were investigated through precise oligomer synthesis having different chains ends (Scheme 1). The first set of PBLG samples terminates with a dimethylamino group (−N­(CH_3_)_2_) at one end and a hydrogen atom at the other. The second set (synthesis reported in ref 16) carries a bulkier end-group consisting of a secondary amine substituted with an *n*-hexyl chain (−NH–(CH_2_)_6_CH_3_) at one end and a hydrogen atom at the other. Because of the low molar masses, chain-end chemistry may influence local mobility, packing efficiency, and the stabilization of specific secondary structures. The chemical structures of both series are shown in Scheme 1.

**Scheme 1:**
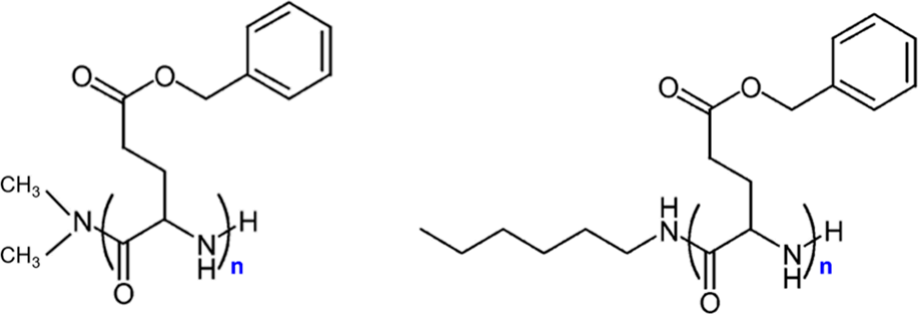
Chemical Structures of Two Poly­(γ-benzyl-l-glutamate) (PBLG) Series Synthesized via Conventional Ring-Opening Polymerization Used in This Study: (Left) PBLG, Terminated with a Dimethylamino Group (−N­(CH_3_)_2_) at One End and a Hydrogen Atom at the Other; (Right) PBLG, Terminated with a Secondary Amine Substituted with an n-Hexyl Chain (−NH–(CH_2_)_6_CH_3_) at One End and a Hydrogen Atom at the Other^a^

a The end-groups result from the different initiators used during synthesis.

The synthesis of the polypeptides was conducted under high-vacuum techniques. Fourier transform infrared (FT-IR) spectroscopy was used to verify the successful completion of the polymerization process. As illustrated in Figure S3, the diminishing NCA peaks (at 1867 and 1844 cm^–1^), along with the emergence of a new absorption band around 1650 cm^–1^ (highlighted in the left green area), corresponding to the amide bond, support this conclusion. Additionally, a distinct peak observed at approximately 1730 cm^–1^ (left green area) indicates the presence of the benzyl ester group, while signals in the 600–800 cm^–1^ range (right green area) are attributed to the vibrations of the aromatic ring, confirming the retention of the protective group, throughout the polymerization process.

The ^1^H NMR spectra of the homopolymers obtained in deuterated chloroform are presented in Figure S4. As observed, in samples with higher molar mass (e.g., containing 100 monomeric units), all peaks correspond to the hydrogen atoms of the polymer. In contrast, for samples with a lower molar mass, additional peaks emerge, which are attributed to the altered chemical environment of the monomers near the DMA initiator. To further investigate this, both the 100-monomeric unit and 7-monomeric unit homopolymer samples were subjected to 2D COSY NMR analysis (Figure S5). The results confirmed the initial hypothesis regarding the peak corresponding to the α-carbon (Ca) of the peptide chain (Figure S4). Specifically, as the polymer length increases, the visibility of the end-group diminishes, resulting in diminishing of the corresponding peaks. Furthermore, the integration of the peaks for both polymers is in accordance with the theoretically predicted values, as presented in Figure S6.

Further characterization of the final homopolymers was conducted using size-exclusion chromatography (SEC). Two SEC setups were used: one in a solution of HFIP/0.05% KTFA at 40 °C and the other in 0.1 LiBr solution of DMF at 60 °C. Initially, the polypeptides were analyzed in DMF. Surprisingly, it was found that polypeptides exhibiting lower degrees of polymerization (DP) showed more than one peak, while at higher DPs, only one peak was evident with diminishing dispersity. In order to examine if the multiple peaks were due to aggregation or side products, we conducted the characterization of the same polymers at the setup in HFIP and at the lowest possible concentrations in order to lower aggregation phenomena. It was found that at HFIP, the polypeptides with rather higher DPs showed one low dispersity peak, while the same polypeptides showed either multiple peaks or one polydisperse peak with weaker side peaks. For example, the SEC eluogram of PBLG_40_ in DMF showed a wide distribution peak (*Đ* = 1.27) with some minor peaks of lower molar mass (Figure 1a). The same polymer showed a low polydispersity peak in HFIP exhibiting *Đ* = 1.08 (Figure 1b).

**Figure 1:**
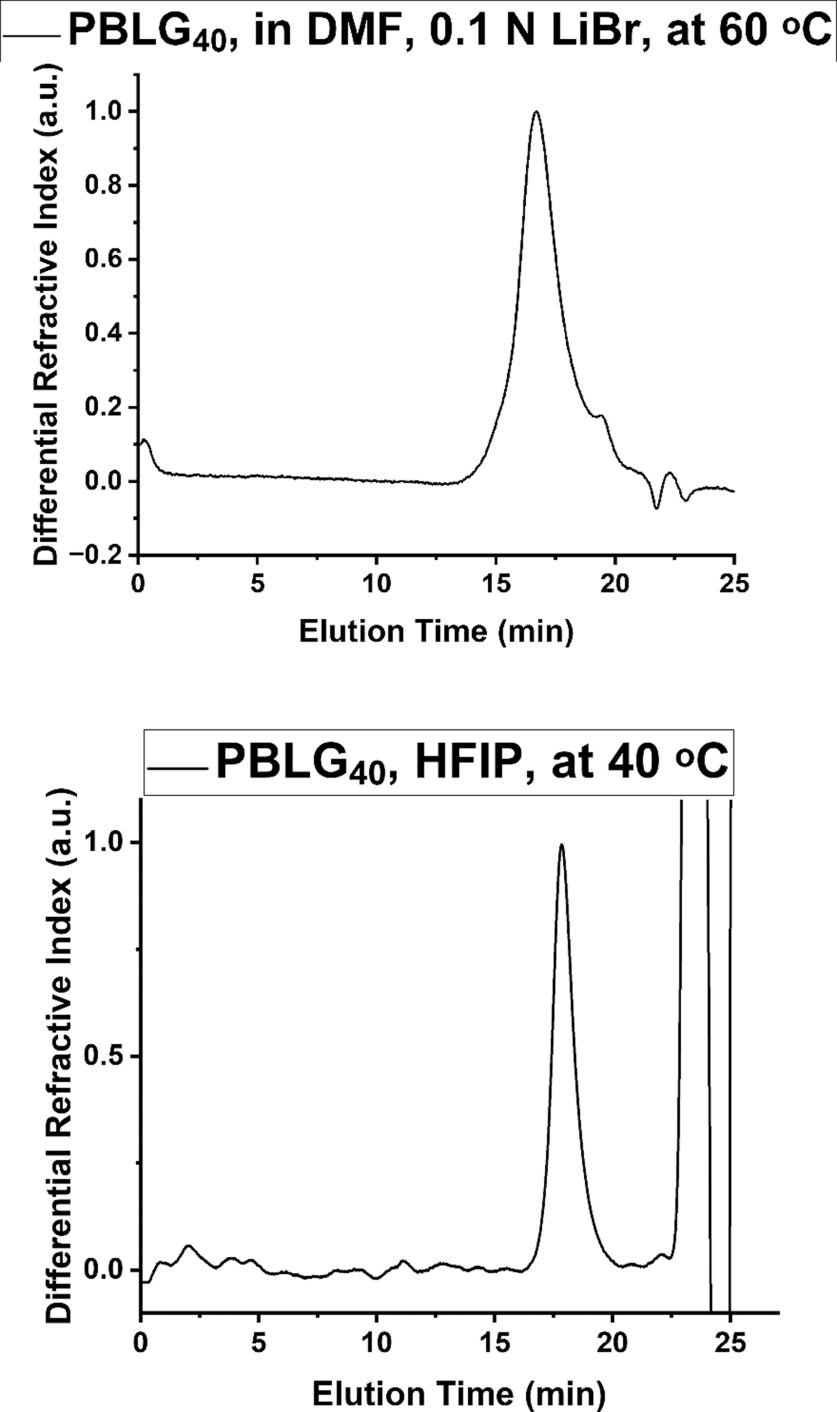
SEC eluograms of (a) PBLG_40_ in DMF, 0.1 LiBr, at 60 °C; (b) PBLG_40_ in HFIP, 0.05% potassium trifluoroacetate at 40 °C.

The noisy baseline in Figure 1b was due to the lowest possible concentration used in order to eliminate aggregation phenomena. The corresponding SEC eluogram of PBLG_100_ in DMF resulted in a low polydispersity peak (*Đ* = 1.07) (Figure S7). For the polypeptides exhibiting lower degrees of polymerization, inconsistent results were obtained by SEC. For example, the SEC eluogram of PBLG_18_ in HFIP resulted in a wider peak (*Đ* = 1.15) exhibiting a minor peak at the higher elution times (Figure S8). The same polypeptide at DMF resulted in multiple peaks (Figure S9).

The degrees of polymerization of all polypeptides were as expected from stoichiometry; however, the multiple peaks obtained for the lower DP polypeptides are due to aggregation phenomena. Since the polypeptides were synthesized by using the same batch of reagents such as NCA, initiator, and DMF, it is evident that the multiple peaks reflect aggregation phenomena and not side products. Therefore, we consider that all the polymers are monodisperse. The purity of the polypeptides exhibiting lower degrees of polymerization cannot be verified by SEC, and possibly, due to polymerization kinetic effects, they could present slightly higher *Đ* values with the effect being higher for polypeptides with lower DPs. The multiple or broadened polydispersity peaks detected in DMF suggest aggregation arising from β-sheet-mediated interactions. Since HFIP acts as a stronger hydrogen bond donor than DMF, it can more effectively disrupt these β-sheet aggregates, although complete dissociation is hindered when the β-sheet content is relatively high.

### 3.2 Thermodynamics and Self-Assembly

The thermodynamics of PBLG depend on molar mass and end-group chemistry (Figures S10, S11, and S12). The DSC traces are shown in Figure 2 (the absolute values of *c*_p_ for three characteristic molar masses are presented in Figure S11). TM-DSC measurements were performed for three oscillation periods, and the characteristic relaxation times were extracted using $\tau_{\alpha ,\mathrm{TM}‐\mathrm{DSC}}=\frac{1}{2\pi f^{\mathrm{TM}‐\mathrm{DSC}}}=\frac{T_{\mathrm{TM}‐\mathrm{DSC}}}{2\pi }$ . These values will be compared later with the DS results. At low degrees of polymerization, *n*, two steps corresponding to two distinct glass temperatures are observed. The lower *T*_g_ is present in all samples, regardless of molar mass. It is attributed to the relaxation of amorphous peptide segments that interrupt the α-helical segments as well as to segments at the chain-ends.^15^ This liquid-to-glass “transition” follows a Fox–Flory dependence (shown in Figure 10). Interestingly, a second, higher *T*_g_ is observed in oligopeptides, especially in the derivative representation of *c*_p_ (Figure 2b). This glass temperature is assigned to the dynamics of segments located in less ordered or completely disordered parts of β-sheets. To our knowledge, this is the first time that a β-sheet-associated *T*_g_ is reported in a completely nonhydrated (hydrophobic) polypeptide. The presence of two *T*_g_s in oligopeptides confirms that the corresponding relaxation processes, here referred to as α and α* segmental dynamics, are both thermodynamically active and contribute to the configurational entropy of the system. Unlike a previous report on PBLG,^36^ we did not observe an irreversible first-order transition from a 7/2 helix to the more thermodynamically stable 18/5 α-helix during the first heating. In the Supporting Information (Figure S10), we include DSC traces of selected PBLG samples over a broader temperature range (up to decomposition). The traces do not show the reported endothermic feature at ∼350 K.

**Figure 2:**
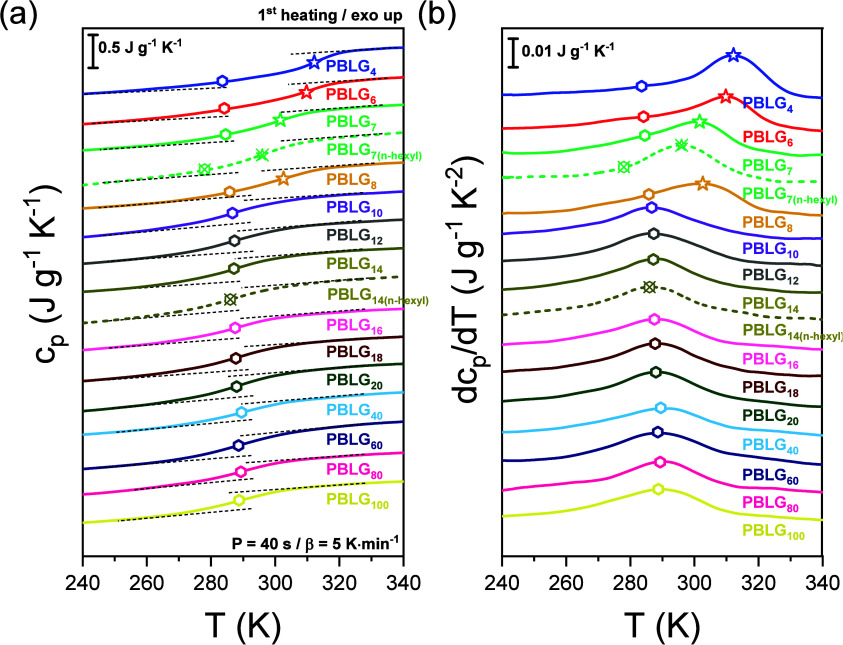
(a) Temperature dependence of the specific (reversing) heat for a series of PBLGs with different molar masses, obtained from TM-DSC at a period of modulation of *P* = 40 s (β = 5 K s^–1^). Dashed lines correspond to the *n*-hexyl terminated samples. Data are shifted for clarity. (b) Derivative of the specific heat capacity with respect to temperature plotted as a function of temperature. Polygons indicate the main liquid-to-glass temperature of PBLG. Star symbols correspond to the additional glass temperature, e.g., the high *T*_g_, evident only in low-molar-mass PBLG.

To quantify the contribution of each secondary structure and to extract the relative α-helical and β-sheet fractions for the oligopeptides, the derivative representation of the *c*_p_ was fitted to a summation of two Lorentzian functions (Figure S12). As discussed, the lower *T*_g_ was assigned to amorphous peptide segments that interrupt the α-helical segments as well as to segments at the chain-ends, while the higher *T*_g_ to the dynamics of segments located in less ordered or completely disordered parts of β-sheets. The relative contribution of each *T*_g_ can be estimated from the integrated areas under each Lorentzian peak (*A*_α‑helix_ and *A*_β‑sheet_, respectively), according to $f_{\alpha ‐\mathrm{helix}}^{\mathrm{DSC}}=\frac{A_{\alpha ‐\mathrm{helix}}}{A_{\alpha ‐\mathrm{helix}}+A_{\beta ‐\mathrm{sheet}}}$ and $f_{\beta ‐\mathrm{sheet}}^{\mathrm{DSC}}=\frac{A_{\beta ‐\mathrm{sheet}}}{A_{\beta ‐\mathrm{sheet}}+A_{\alpha ‐\mathrm{helix}}}$ . The results are summarized in Table 1. They reveal good agreement with the WAXS and NMR results (discussed below with respect to Figure 4). Interestingly, a significant distinction is evident between the oligopeptide PBLG_7(n‑hexyl)_ and its analog PBLG_7_ terminated by the dimethylamino group. Specifically, PBLG_7(n‑hexyl)_ exhibits a substantially higher β-sheet fraction (Figure S12d), nearly twice that of α-helices. Quantitatively, in DSC, the *n*-hexyl-terminated sample consists of approximately 64% β-sheets and 36% α-helices. In comparison, the dimethylamino-terminated PBLG_7_ has approximately 62% α-helices and ∼38% β-sheets. These results highlight the critical effect of end-groups on the secondary structure formation. On the other hand, for PBLG_14(n‑hexyl)_ and its dimethylamino analog, it is difficult to identify two distinct glass temperatures in the thermodynamic traces. Overall, the results indicate that increasing *n* promotes the stabilization of α-helices, whereas β-sheets are formed only at low *n*. In the dimethylamino-terminated samples, however, α-helices are the predominant secondary structure, as $f_{\alpha ‐\mathrm{helix}}^{\mathrm{DSC}}>f_{\beta ‐\mathrm{sheet}}^{\mathrm{DSC}}$ , for all molar masses investigated. We will return to this point following the WAXS and NMR investigations.

**Table 1:** Relative Fractions of α-Helices and β-Sheets for the Investigated PBLG, as Obtained from DSC and WAXS, and Absolute Fractions of α-Helices and β-Sheets, as Obtained from NMR (at 320 K)

	DSC		WAXS		NMR	
samples	*f*_β‑sheet_	*f*_α‑helix_	*f*_β‑sheet_	*f*_α‑helix_	*f*_β‑sheet_	*f*_α‑helix_
PBLG_4_	0.44	0.56	0.40	0.60	0.44	0.56
PBLG_6_	0.40	0.60	0.40	0.60	0.41	0.59
PBLG_7_	0.38	0.62	0.40	0.60		
PBLG_7(n‑hexyl)_	0.64	0.36	1	0	0.81	0.19
PBLG_8_	0.36	0.64	0.40	0.60	0.34	0.66
PBLG_10_			0.37	0.63		
PBLG_12_			0.36	0.64		
PBLG_14_			0.35	0.65	0.21	0.79
PBLG_14(n‑hexyl)_			0.52	0.48	0.57	0.43
PBLG_16_			0.33	0.67		
PBLG_18_			0.28	0.72		
PBLG_20_			0.30	0.70		
PBLG_40_			0	1	0.03	0.97
PBLG_60_			0	1		
PBLG_80_			0	1		
PBLG_100_			0	1		

The self-assembly of PBLG was subsequently investigated as a function of the degree of polymerization by employing WAXS and ^13^C solid-state NMR. The WAXS patterns for four representative dimethylamino terminated samples, along with their *n*-hexyl terminated analogs, are presented in Figure 3 (and Figure S13 for the remaining samples). Two distinct groups can be identified: (i) short to intermediate oligopeptides (*n* = 4, 6, 7, 8, 10, 12, 14, 16, 18, and 20) that consist of both secondary structures and (ii) polypeptides (*n* = 40, 60, 80, and 100) where only the α-helical secondary structure is present. In the WAXS curves, all dimethylamino-terminated PBLG samples exhibit, at low scattering vectors, a set of strong Bragg reflections with characteristic ratios of 1:3^1/2^:4^1/2^, with the primary peak at *q* ∼ 4.7 nm^–1^ (intercylinder distance of ∼1.54 nm), indicating the hexagonal packing of α-helices. This structure of PBLG, known as the nematic-like paracrystalline form C, describes a periodic packing of α-helices in the direction lateral to the chain axis with a nematic-like paracrystalline order.^16,36^ In the polypeptides, an additional sharp reflection is observed at higher *q* (= 12.6 nm^–1^) corresponding to the helical pitch of 0.5 nm along the chain axis. Oligopeptides show an additional Bragg reflection at *q* = 3.7 nm^–1^ (*d* = 1.7 nm), corresponding to the lamellar spacing or the intersheet spacing of β-sheets. The PBLG_7(*n*‑hexyl)_ displays reflections exclusively associated with the β-sheet structure, consistent with its high β-sheet fraction (∼80% according to NMR results). In addition, *n*-hexyl-terminated oligopeptides show an additional peak at *q* = 13.4 nm^–1^, corresponding to an interstrand spacing of 0.47 nm between adjacent peptide chains within the β-sheets. The broad peak (amorphous halo or van der Waals peak) at around 14 nm^–1^, evident in all peptides, is assigned to correlations between the side-group atoms and contributions from the amorphous PBLG regions. Disordered segments along the PBLG chains coexist with the ordered configurations identified as α-helices or β-sheets. In fact, amorphous segments have a distinct dynamic signature in DS (see Figure 11 below).

**Figure 3:**
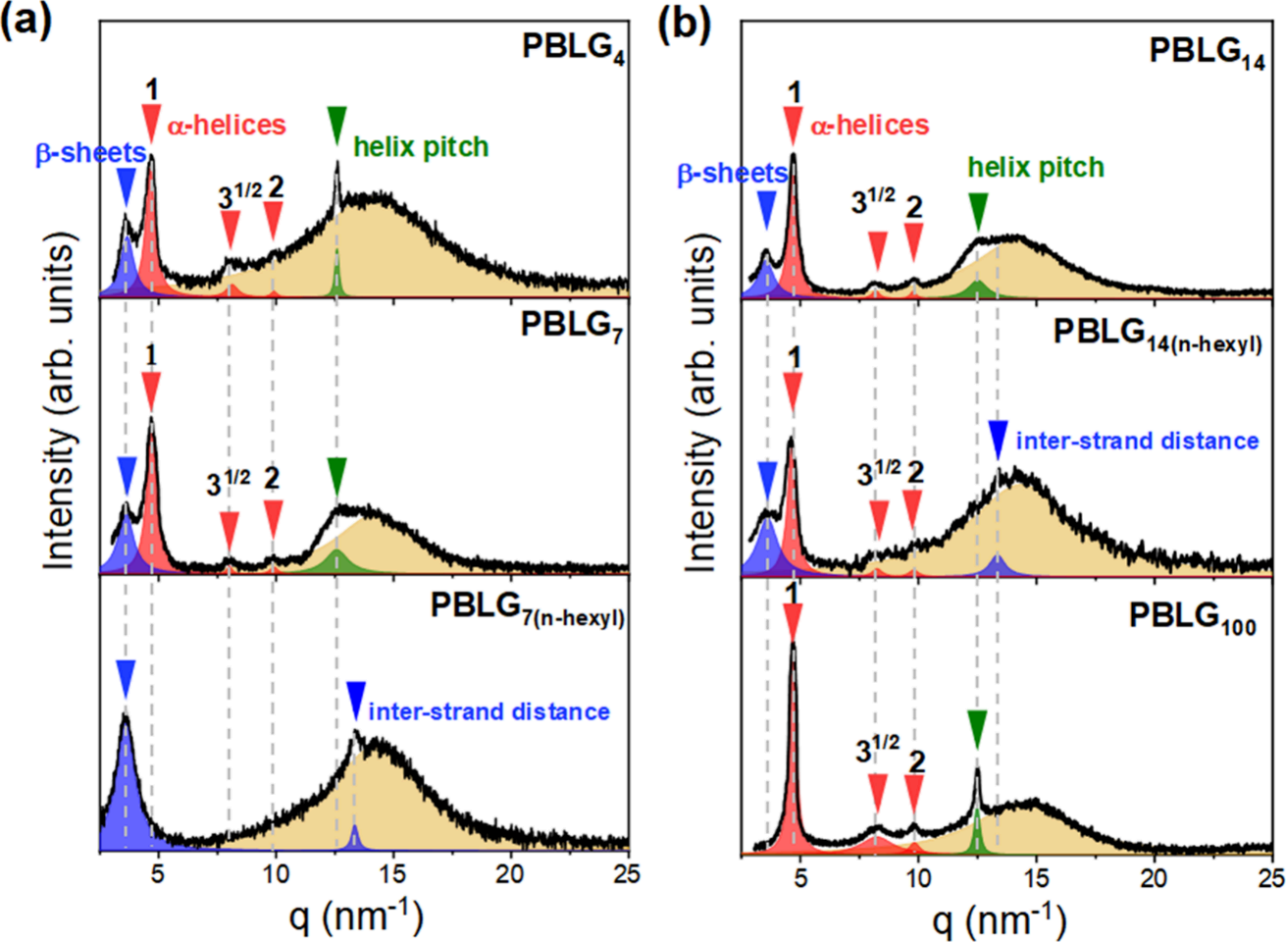
WAXS patterns of the investigated samples (a) top: PBLG_4_, middle: PBLG_7_, bottom: PBLG7_(n‑hexyl)_ and (b) top: PBLG_14_, middle: PBLG_14(n‑hexyl)_, bottom: PBLG_100_. Blue arrows correspond to the lamellar spacing of the β-sheet secondary structure and the interstrand distance of β-sheets. Red arrows indicate Bragg reflections of the hexagonally packed α-helices, while green arrows give the reflection corresponding to the pitch of the α-helix. The amorphous halo is indicated in yellow.

In WAXS, the relative fractions of α-helices and β-sheets were determined from the integrated intensities of respective Bragg peaks. This analysis assumes that there are only two ordered populations (α-helices and β-sheets) contributing to the scattering. Prior to integration, all curves were corrected for the background. The values are summarized in Table 1 and also presented in Figure 4a. The dimethylamino-terminated PBLGs display a consistently higher α-helical fraction over all chain lengths. The evolution of both $f_{\alpha ‐\mathrm{helix}}^{\mathrm{WAXS}}\;\mathrm{and}\;f_{\beta ‐\mathrm{sheet}}^{\mathrm{WAXS}}$ as a function of the degree of polymerization can be described by a generalized sigmoidal function as *y* = *A* – (*A* – *B*) $e^{-{\left(kx\right)}^{d}}$ , where *A* is the final (asymptotic) value, *B* is the initial value, *k* is a rate parameter, and *d* is a shape parameter controlling the sharpness of the “transition”. The fit to the α-helical fraction data yields the equation *f*_α‑helix_ = 1 – 0.4 e^−(0.04*n*)^3.2^^, while for the β-sheet fraction data, *f*_β‑sheet_ = 0.4 (1 – e^−(0.05*n*)^−3.6^^). Both shape parameters indicate a progressive transformation to the secondary fractions for *n* > 20.

**Figure 4:**
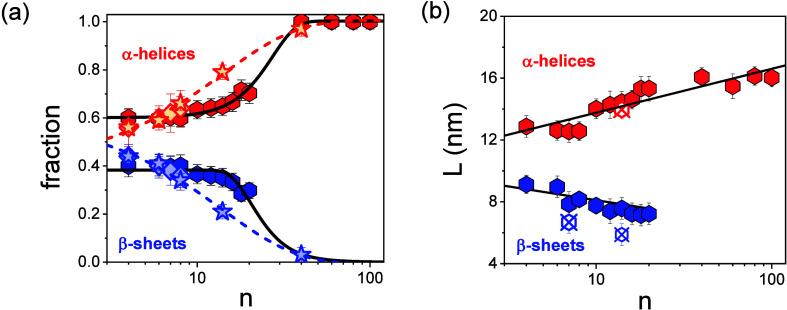
(a) Relative (TM-DSC: rhombi, WAXS: hexagons) and absolute (NMR: stars) fractions of α-helices (red) and β-sheets (blue) of the dimethylamino-terminated samples as a function of the degree of polymerization. Black lines are sigmoidal fits to the WAXS results (see the text). Red and blue lines represent linear fits to the NMR data. (b) Coherence length of the hexagonally packed α-helices (red) and of the lamellar assembly of the β-sheets (blue) over molar mass. Crossed symbols give the results for the n-hexyl-terminated oligopeptides. Black lines are to guide the eyes.

A key parameter characterizing the self-assembly of the secondary structures is the lateral coherence length of their ordered domains in their lamellar β-sheets and the hexagonal packed α-helices. This parameter can be extracted from the fwhm of the corresponding primary Bragg reflections, as *L* = 2π/*w* (where *w* = fwhm). The calculated lengths are presented in Figure 4b. The results, seen together with Figure 4a, reveal a connection between the increasing (decreasing) fraction of α-helices (β-sheets) and the lateral coherence of the respective domains.

The structural behavior was further investigated by ^13^C solid-state NMR. Representative samples, i.e., PBLG_4_, PBLG_6_, and PBLG_8_ from the oligomers, PBLG_14_ from the intermediate *n*, and PBLG_40_ from the polypeptides, were selected to investigate the evolution of the secondary structure with the chain length through the characteristic chemical shifts of C_α_ and C=O carbon sites. Corresponding NMR spectra are shown in Figure 5. As well-established in the literature,^27^ each secondary structure exhibits resonances at distinct carbon chemical values for C=O and C_α_ sites. α-Helical structures can be identified by the resonances at chemical shifts δ ∼176 ppm and δ ∼58 ppm, corresponding to the amide C=O and C_α_ carbon, respectively. In contrast, β-sheets lead to upfield shifted resonances of the amide C=O and C_α_ carbons at δ ∼172 ppm and δ ∼53 ppm, respectively. In addition, different from WAXS, the C_α_ carbon next to the NH_2_ chain-end can be distinguished from the other C_α_ carbon sites of the β-sheet and is observed as a separate resonance at ∼49 ppm.

**Figure 5:**
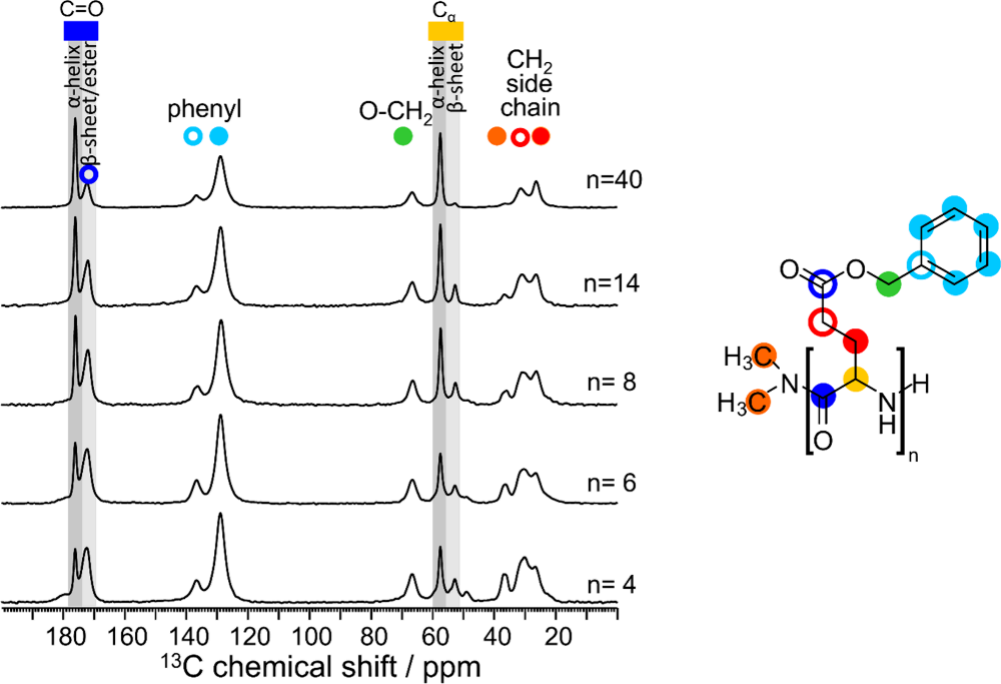
^13^C solid-state NMR spectra of the dimethylamino-terminated samples: PBLG_4_, PBLG_6_, PBLG_8_, PBLG_14_ and PBLG_40_. The highlighted areas refer to the chemical shifts used for the calculation of the absolute fractions of peptide secondary structures. The molecular structure of the repeat unit is also shown, including the color scheme employed for assignment purposes.

The calculated absolute fractions of the α-helices and β-sheets (including the contribution of the chain-ends) are presented in Table 1, together with the corresponding DSC and WAXS data. In NMR, the fractions were calculated based solely in the C_α_ carbon resonances. The NMR spectra show a progressive increase in the intensity of the α-helical resonances with increasing *n*, accompanied by a corresponding decrease in the β-sheet signals. For the dimethylamino-terminated samples, this trend can be described by the dependencies *f*_α‑helix_ = 0.28 + 0.43 × log *n* and *f*_β‑sheet_ = 0.57 – 0.33 × log *n*. Notably, β-sheet-associated resonances decrease substantially and eventually disappear at *n* ≥ 40, indicating that β-sheets become unstable in this regime. For the *n*-hexyl-terminated samples, although the available data are limited, the observed fits are indicative of a similar trend. For intermediate *n*, as expected, both secondary structure signals are evident, revealing coexisting α-helical and β-sheet structures. These observations are in good agreement with the structural insights from WAXS and the thermodynamic results obtained from TM-DSC. The combined results of the three techniques are summarized in Figure 4a. It depicts the secondary structure evolution with molar mass: from β-sheet-rich domains at low *n* to α-helical structures at higher *n*. This brings forward a question on the thermodynamic stability of secondary structures in PBLG. Between the different secondary structures, the α-helix is the preferred one (the one with the lowest energy) as it is the sole secondary structure for the higher molar masses. It is only in the oligopeptides that the criteria for stable helices (e.g., 18/5 of 7/2) are not satisfied, and β-sheets are formed. This is understandable, as intramolecular hydrogen bonds can be formed first giving rise to α-helices, whereas β-sheets require extensive intermolecular hydrogen bonds of higher cooperativity.

How do these results depend on end-group chemistry? To further probe the role of end-group chemistry and its influence on the self-assembly, detailed ^13^C solid-state NMR spectra, together with the corresponding ^1^H NMR spectra, were acquired for PBLG_14_ and its n-hexyl-terminated analog, PBLG_14(n‑hexyl)_ (Figure S14). Although both samples share the same average degree of polymerization, the nature of their end-groups is different. PBLG_14_ features a hydrogen atom at one end and a dimethylamino group (−N­(CH_3_)_2_) at the other end, while PBLG_14(n‑hexyl)_ has a hydrogen atom at one terminus and a longer, bulkier end-group consisting of a secondary amine substituted with an *n*-hexyl chain (−NH–(CH_2_)_6_CH_3_) at the other end. The ^13^C NMR spectra reveal distinct resonances in the upfield region, corresponding to the terminal carbons of these end-groups, along with the two carbons of the side group. These assignments are further supported by the ^1^H NMR spectra, which display the corresponding proton signals of the terminal moieties. PBLG_14_ has predominantly α-helical structures, whereas PBLG_14(n‑hexyl)_ shows a majority of β-sheets in agreement with the DSC and WAXS results (Table 1). Hence, chain-end chemistry contributes significantly to the stabilization of a certain secondary structure. We can envision that the alkyl chain in the *n*-hexyl-terminated oligopeptides results in a local segregation that favors the formation of β-sheet structures.

### 3.3 Molecular Dynamics

#### 3.3.1 Solid-State NMR

Important insights into the dynamics of PBLG can be obtained by examining the temperature dependence of the NMR spectra, shown in Figure 6. As can be seen from the evolution of the C_α_ resonances of PBLG_6_ and PBLG_14_, the α-helical signal (δ ∼58 ppm) exhibits a significant broadening and a decrease in intensity with increasing temperature. This behavior reflects a temperature-induced softening of the helical segments, especially at the ends of the helices where they are connected to more disordered or amorphous segments. As the system approaches the segmental (α) process (the relative timescales are presented below in Figure 9), the segmental motions fall within the dynamic window of NMR. These thermally activated motions reduce the local rigidity of helices, leading to a broadening of resonances and to a reduction in signal intensity. On the contrary, the β-sheet resonances remain sharp and even increase with temperature (e.g., in PBLG_14_, Figure 6b). The absence of a similar weakening or dynamic broadening implies that the segmental dynamics, hereafter referred to as the α* process, in β-sheet structures are not thermally activated in the studied temperature range up to the frequency (*f* ∼ 10^5^ Hz) needed to average local NMR interaction. Hence, the results of the *T*-dependent NMR study suggest that, up to the highest measurement temperature (*T* = 370 K), the dynamics of β-sheets remain practically frozen on the NMR timescale.

**Figure 6:**
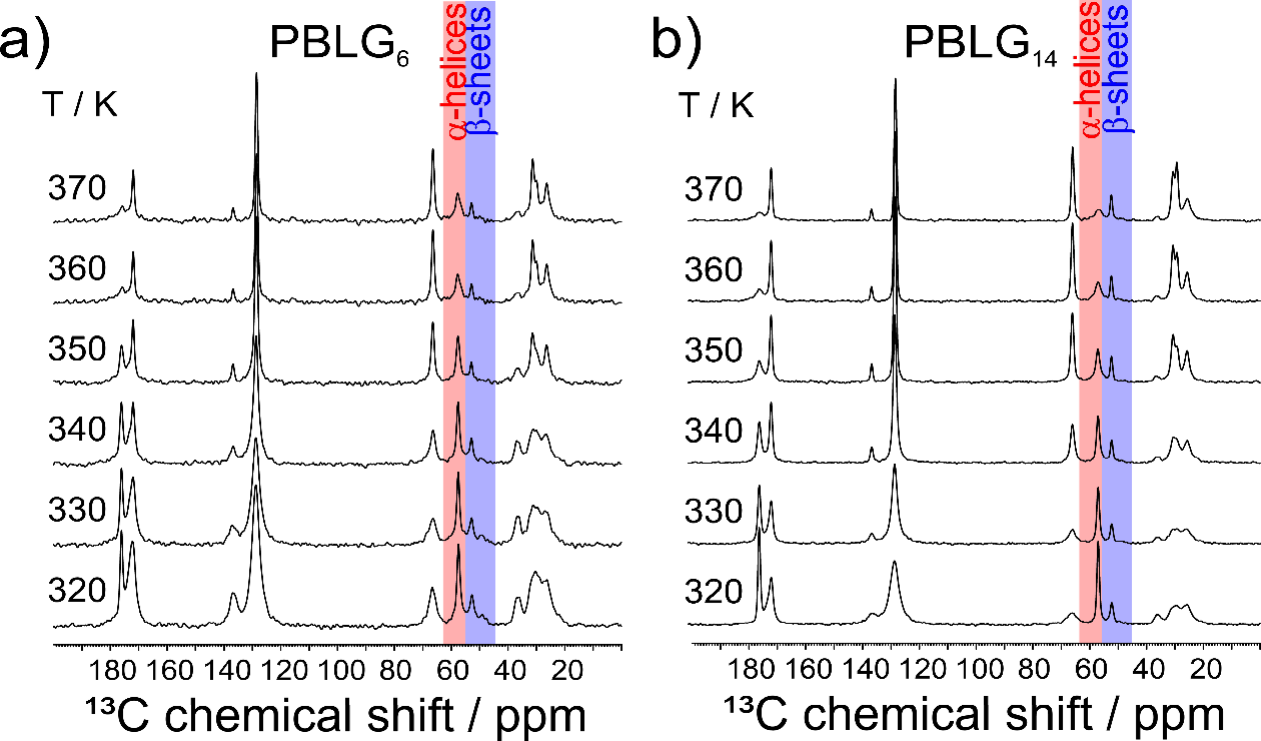
^13^C solid-state NMR spectra of (a) PBLG_6_ and (b) PBLG_14_ as a function of temperature. The highlighted areas refer to the chemical shifts used for the calculation of the absolute fractions of α-helices and β-sheets.

Additional site-specific information on the rigidity and differences in local molecular mobility of the β-sheet and α-helical segments can be obtained by site-specific ^1^H–^13^C REPT-HDOR spinning sideband pattern measurements as a function of temperature for PBLG_14_ (Figure S15 and Figure 7).^30,31^ These experiments provide access to the motional averaged dipolar coupling constants, ⟨*D*_CH_(*t*)⟩*_t_*, from which the local dynamic order parameter, *S*, is derived. The parameter *S*,^37^ defined in terms of the time-averaged second-order Legendre polynomial, quantifies the residual motional anisotropy of a given molecular segment:

$$S_{\mathrm{CH}}={\left\langle \frac{1}{2}\left(3\cos^{2}\theta_{\mathrm{CH}}\left(t\right)-1\right)\right\rangle }_{t}=\frac{{\left\langle D_{\mathrm{CH}}\left(t\right)\right\rangle }_{t}}{D_{\mathrm{CH},\mathrm{static}}}$$ 3

**Figure 7:**
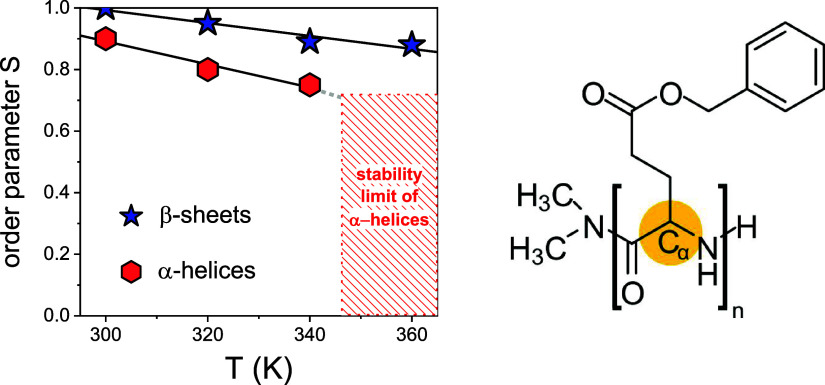
(left) Temperature dependence of the dynamic order parameter *S* for the C_α_–H bonds in the two secondary structures, α-helices and β-sheets. *S* = 0 indicates isotropic motion, while *S* = 0.7 indicates strongly anisotropic motion with a Gaussian orientation distribution of 73° width at half height. (right) The given order parameter *S* has been determined for the CH bond of the C_α_ position highlighted in the molecular structure for clarity.

A value of *S* = 1 indicates complete rigidity, while lower values reflect increasing amplitudes of molecular motion on the timescale of the NMR experiment. As the signals of C_α_ sites in α-helices/β-sheets are well-resolved in the ^13^C NMR spectra, *S* can be calculated for each secondary structure (Figure S15). For the β-sheets, *S* remains nearly constant over the entire temperature range studied here, with only a small decrease from 1.0 (*D* = 22.7 kHz) at 300 K to 0.88 (*D* = 19.7 kHz) at 360 K. This slight decrease reflects the onset of limited local molecular fluctuations but overall confirms that β-sheet structures remain highly rigid and dynamically arrested up to 360 K. These findings are consistent with the glassy behavior of β-sheets observed by DS (see below). In contrast, the α-helical segments show a significantly different response. Starting from a slightly lower *S* value of 0.90 (*D* = 20.3 kHz) at 300 K, the order parameter gradually decreases, reaching 0.75 at 340 K. This continuous decrease indicates increasing local mobility and partial dynamic softening of the helical structures. At 360 K, the REPT-HDOR signal of α-helical segments is not observed due to a substantial reduction of the residual dipolar coupling and significantly shorter ^13^C T_2_ times already observed as increasing line width in the temperature-dependent ^13^C CP MAS NMR spectra shown in Figure 6. These results indicate that the α-helical structures in PBLG become unstable at 360 K due to the onset of large-amplitude segmental dynamics and the lifetimes of the α-helical structures are too short to be probed on a ms timescale by the REPT-HDOR NMR measurement. As we will show bellow with DS, this reflects the onset of the segmental (α) process, e.g., the segmental motion in α-helical peptides. The distinct *S*(*T*) profiles displayed in Figure 7 quantitatively demonstrate this divergence in mobility: while β-sheets remain largely immobilized, α-helices undergo a temperature-driven relaxation into a dynamically softened state.

#### 3.3.2 Dielectric Spectroscopy

The structural characterization (WAXS and solid-state NMR) of PBLG as a function of the degree of polymerization identified distinct self-assembly motifs. In addition, temperature-dependent solid-state NMR identified dynamic changes in the NMR frequency, especially for the α-helices, whereas β-sheets were found to be “glassy” up to about 360 K. Although NMR could identify glassy vs mobile segments, it was not possible to extract the timescales of respective motions. This inherent difficulty of NMR can be surpassed by employing dielectric spectroscopy. Here, we discuss the molecular dynamics as a function of temperature and pressure by employing DS.

First, we discuss the molecular dynamics for the different molar masses (Figure S16 and Figure 8). Figure 8 shows a three-dimensional plot of the derivative of dielectric permittivity as a function of frequency and temperature for three representative samples: PBLG_6_, PBLG_7(n‑hexyl)_, and PBLG_60_. Starting from lower temperatures, all samples, regardless of the degree of polymerization and end-group, exhibit the α process. This process originates from the amorphous segments that interrupt the α-helices, as well as from segments at the chain-ends.^14–16,36^ It corresponds to the lowest glass temperature detected in TM-DSC. For peptides with low and intermediate molar masses (*n* ≤ 20) that stabilize both α-helical and β-sheet secondary structures, two additional relaxations are evident. The first is the α* process, attributed to the dynamics of segments located in less ordered or completely disordered parts of β-sheets. This is the first experimental observation of a segmental relaxation directly associated with the β-sheets (Figure 8a,b). Interestingly, the α* process occurs at lower frequencies, i.e., longer timescales, than the α process at the same temperature (Figure 9). This indicates that the associated motions are significantly more cooperative. The second process, termed “slow” process, is associated with a global relaxation of β-sheets. This process will be discussed in detail below, in the context of its dielectric strength.

**Figure 8:**
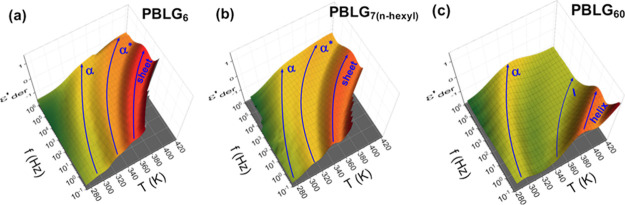
Three-dimensional representation of the derivative of dielectric permittivity as a function of frequency and temperature, for (a) PBLG_6_, (b) PBLG_7(n‑hexyl)_, and (c) PBLG_60_. Three processes are visible in each case; for the oligopeptides, PBLG_6_, and PBLG_7(n‑hexyl)_, the indicative lines correspond to the α process, the α* process, and the slow β-sheet process, while for PBLG_60_, the lines give the α process, a weak intermediate process, and the slower relaxation of α-helices.

**Figure 9:**
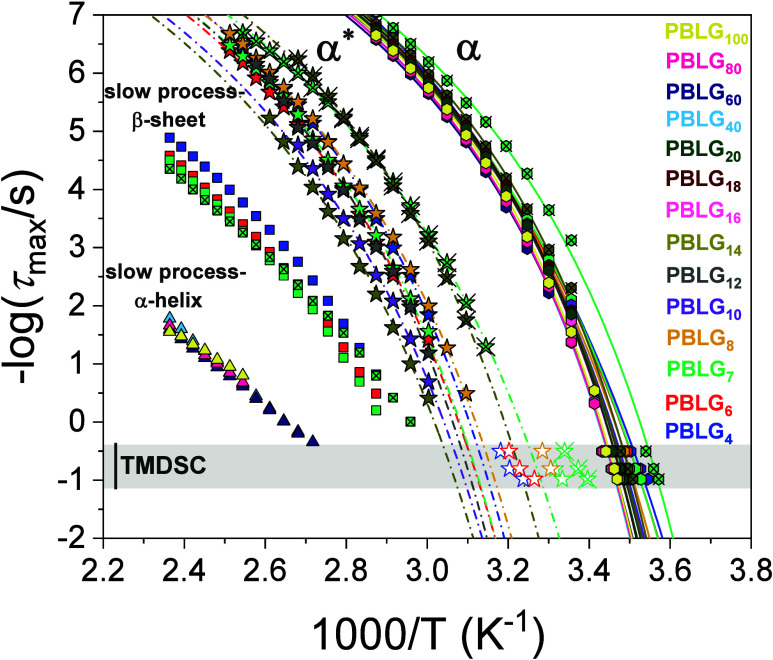
Characteristic relaxation times as a function of the inverse temperature of PBLGs with different degrees of polymerization: PBLG_4_ (blue), PBLG_6_ (red), PBLG_7_ (green), PBLG_8_ (orange), PBLG_10_ (purple), PBLG_12_ (gray), PBLG_14_ (dark yellow), PBLG_16_ (pink), PBLG_18_ (wine), PBLG_20_ (dark green), PBLG_40_ (light blue), PBLG_60_ (navy), PBLG_80_ (magenta), and PBLG_100_ (yellow). Crossed symbols correspond to PBLG_7(n‑hexyl)_ (green) and PBLG_14(n‑hexyl)_ (dark yellow). Hexagons represent the segmental α process, stars the segmental α* process, squares the “slow” β-sheet process, and triangles the “slow” α-helical process (see the text). TM-DSC data are also shown. The lines represent fits to the VFT equation (parameters are summarized in Table 2).

For polypeptides (40 ≤ *n* ≤ 100) that exclusively adopt α-helical structures, the results are consistent with the previous studies on PBLG (Figure 8c).^14–16,36^ At higher *T*, a slower and intense process is observed. It is assigned to the relaxation of macrodipoles in the helical parts, the so-called slow α-helix process. According to the “defected helix” model,^15^ the α-helix can be conceptualized as consisting of distinct “ideal” helical parts of uniform correlation length, denoted as ξ_helix_, which are capable of rotating on the surface of a cone at a defined angle θ. Due to the hexagonal packing of helices, it is assumed that each part rotates independently, while the axes of the rotational cones remain parallel. The dipole moment associated with each helical part is μ = 3.4 *D* (ξ/0.15 nm), where 0.15 nm is the helix length per repeat unit. This model allows the estimation of the ξ_helix_ from the dielectric strength of the slow helical process as $\Delta \varepsilon =\frac{N_{A}\rho }{3\varepsilon_{0}kTM_{o}}{\left(3.4\;\mathrm{Debye}\right)}^{2}\left(\xi /0.15\;\mathrm{nm}\right)\;\sin^{2}\theta$ . X-ray scattering from oriented fibers provides an upper value for θ, which is then used to estimate the helical correlation length. This value is approximately 2 nm, significantly smaller than the theoretical length of an “ideal” helix. This suggests that α-helices consist of broken helices rather than behaving as a single rigid unit.^15^

The extracted relaxation times for the aforementioned processes are presented in the Arrhenius plot of Figure 9. The α and α* processes conform to the usual Vogel–Fulcher–Tammann (VFT) equation

$$\tau =\tau_{o}^{\#}\;\exp\left(\frac{B}{T-T_{o}}\right)$$ 4

where $\tau_{o}^{\#}$ is the relaxation time in the limit of very high temperatures, *B* is the activation parameter, and *T*_o_ is the “ideal” glass temperature. The VFT parameters of the two segmental processes in the oligopeptides and the polypeptides with the different terminated groups are summarized in Table 2.

**Table 2:** VFT Parameters for the Segmental α and α* Processes, along with the Corresponding *T*_g_ Values Extracted from DS (at τ = 100 s) and DSC

	sample	–log(τ_ο_/s)	*B* (K)	*T*_0_ (K)	*T*_g_^DS^ (K)	*T*_g_^DSC^ (K)
α process	PBLG_4_	–14^a^	2015 ± 20	225 ± 1	279 ± 1	284 ± 3
	PBLG_6_	–13.4 ± 0.3	1800 ± 85	229 ± 2	280 ± 1	284 ± 3
	PBLG_7_	–13.2 ± 0.2	1710 ± 65	230 ± 1	281 ± 1	285 ± 3
	PBLG_7(*n*‑hexyl)_	–12	1250 ± 15	238 ± 1	277 ± 1	278 ± 3
	PBLG_8_	–12.6 ± 0.1	1490 ± 45	237 ± 1	282 ± 1	286 ± 3
	PBLG_10_	–12.3 ± 0.5	1384 ± 55	241 ± 3	283 ± 1	287 ± 1
	PBLG_12_	–12^a^	1265 ± 5	244 ± 1	284 ± 1	287 ± 1
	PBLG_14_	–12^a^	1280 ± 10	244 ± 1	283 ± 1	287 ± 1
	PBLG_14(*n*‑hexyl)_	–12^a^	1225 ± 15	244 ± 1	282 ± 1	286 ± 1
	PBLG_16_	–12^a^	1235 ± 15	246 ± 1	284 ± 1	288 ± 1
	PBLG_18_	–12^a^	1210 ± 20	247 ± 1	284 ± 1	288 ± 1
	PBLG_20_	–12^a^	1235 ± 10	246 ± 1	284 ± 1	288 ± 1
	PBLG_40_	–12^a^	1260 ± 20	247 ± 1	286 ± 1	289 ± 1
	PBLG_60_	–12^a^	1295 ± 20	245 ± 1	285 ± 1	289 ± 1
	PBLG_80_	–12^a^	1265 ± 15	246 ± 1	286 ± 1	289 ± 1
	PBLG_100_	–12^a^	1240 ± 10	247 ± 1	285 ± 1	289 ± 1
α* process	PBLG_4_	–12^a^	1690 ± 10	261 ± 1	313 ± 1	312 ± 1
	PBLG_6_	–12^a^	1795 ± 15	260 ± 1	316 ± 1	310 ± 1
	PBLG_7_	–12^a^	1735 ± 25	262 ± 1	316 ± 1	302 ± 1
	PBLG_7(*n*‑hexyl)_	–12^a^	1760 ± 15	245 ± 1	301 ± 1	296 ± 1
	PBLG_8_	–12^a^	1725 ± 10	258 ± 1	312 ± 1	302 ± 1
	PBLG_10_	–12^a^	1920 ± 30	259 ± 1	319 ± 1	
	PBLG_12_	–12^a^	1700 ± 35	265 ± 2	318 ± 1	
	PBLG_14_	–12^a^	1980 ± 45	260 ± 2	321 ± 1	
	PBLG_14(*n*‑hexyl)_	–12^a^	1570 ± 20	257 ± 1	305 ± 1	

a Value held fixed.

As a next step, we examine the dependence of the glass temperatures on molar mass. The *T*_g_s associated with the α and α* segmental processes, as determined by both TM-DSC and DS, are plotted as a function of the degree of polymerization *n* in Figure 10. The *T*_g_ of the α process increases with an increasing chain length, following an approximately Fox–Flory dependence, suggesting the influence of chain-ends on the segmental dynamics. Despite a more limited data set, the *T*_g_ of the α* process shows a similar trend. Both processes are attributed to the dynamics of amorphous segments, especially those located at the chain-ends or in disordered regions in-between the more ordered domains. However, the key difference lies in their structural environment: the α* process occurs at higher temperatures, suggesting that these amorphous segments are subject to additional constraints. The reduced mobility of segments associated with the α* process in β-sheets is likely to associate with h.b. defects between different chains in their β-sheet secondary structure.

**Figure 10:**
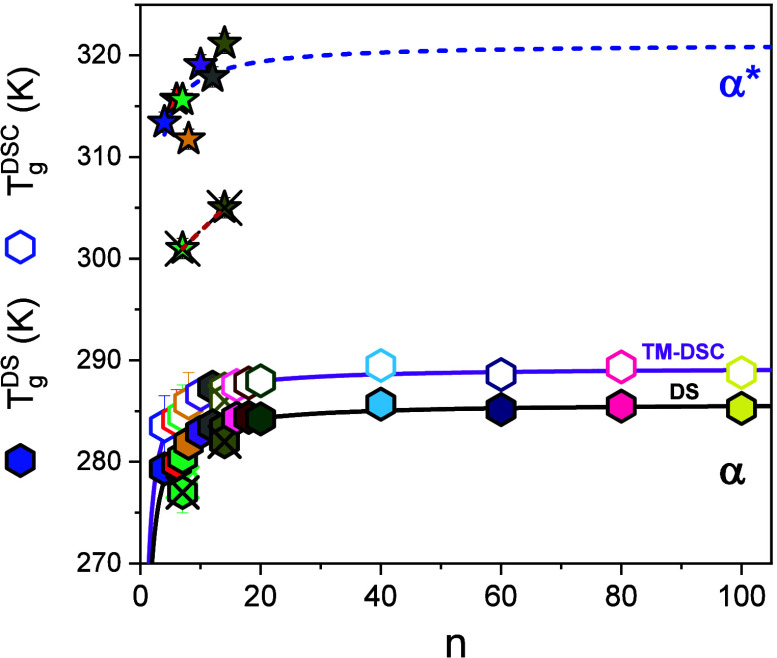
Liquid-to-glass temperatures of the segmental α process (hexagons) and the segmental α* process (stars) as a function of the molar mass. Crossed symbols correspond to the *n*-hexyl-terminated oligopeptides. Filled symbols refer to DS (*T*_g_ defined at τ = 100 s) and open symbols to TM-DSC. Lines represent fits to the Fox–Flory equation.

Interestingly, the *n*-hexyl-terminated samples exhibit lower *T*_g_s, as compared to the dimethylamino-terminated ones, both for the α-helical (α) and β-sheet (α*) secondary structures.

We now turn to the analysis of the “slow” process, observed at elevated temperatures in PBLG with low and intermediate molar masses. In α-helical polypeptides, this process is attributed to the relaxation of macrodipoles in the α-helical parts. The ratio of the dielectric strength of the “slower” process associated with the secondary structure relaxation (α-helix or β-sheet) to the dielectric strength of the corresponding segmental process (α or α* process, respectively) is shown in Figure 11a as a function of molar mass. For high *n* (≥40), the ratio is Δε_α‑helix_/Δε_α_ ∼ 4, consistent with the relaxation of α-helical parts, with an effective dipole moment (macrodipole) larger than that of the segmental process. Conversely, for low *n* (4 ≤ *n* ≤ 10), the ratio is Δε_β‑sheet_/Δε_α*_ ∼ 2. This is again suggestive of a relaxation exceeding a repeat unit in the β-sheet configuration. Such a process can result from the parallel β-sheet structures, where the vectorial sum of individual amino acid residues in a β-sheet creates a macrodipole (Figure 11b). With respect to macrodipoles, β-sheets can be considered as type-B polymers according to the Stockmayer classification (α-helical peptides are of type-A).^38^ Type-B polymers have rigid dipoles perpendicular to the chain and give rise to normal modes. This was evidenced in functionalized poly-*p*-phenylenes bearing ultrastrong dipoles perpendicular to the backbone.^39^

**Figure 11:**
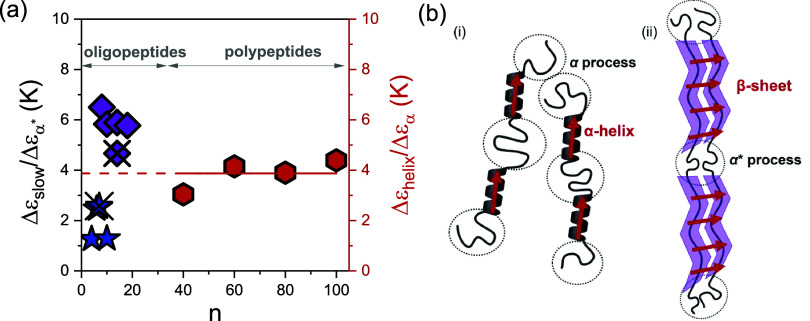
(a) Dielectric strength of the slow process (blue) normalized to the dielectric strength of the α* process and the α-helix process (red) normalized to the dielectric strength of the α process, for oligopeptides and polypeptides, as a function of the degree of polymerization. The case of polypeptides with intermediate *n*, where the dielectric strength of the slow process (purple) is normalized to the dielectric strength of the α* process, is also indicated. Crossed symbols correspond to the *n*-hexyl-terminated oligopeptides. (b) Schematic representation of two PBLG chains forming (i) α-helices and (ii) a parallel β-sheet. The red arrows indicate respective macrodipoles. The dotted circles indicate regions where amorphous segments interrupt the α-helices/β-sheets, as well as amorphous segments at the chain-ends.

The dipole moments of polypeptides with alanine and glycine in β-strands have recently been discussed.^22^ They were used as building blocks to predict the macrodipole moment of a β-sheet. Furthermore, it was shown that the dipole moment of an amino acid residue in a β-sheet is smaller than in an α-helix, as experimentally observed. The effect was discussed in terms of a smaller polarization caused by the interstrand hydrogen bonding in a β-sheet as compared to that in an α-helix. Interestingly, in the case of peptides with intermediate degrees of polymerization (12 ≤ *n* ≤ 20), we observe a significantly higher ratio, Δε_slow_/Δε_α*_ ∼ 5. The higher value suggests that the slow process likely arises from a convolution of both β-sheet and α-helical macrodipoles. This interpretation is further supported by the dielectric loss data presented in Figure S16.

A parameter that provides insight into the dynamic behavior of the β-sheets versus the α-helices is the fragility of the corresponding segmental process. The fragility or steepness index, *m*,^40^ is defined as ϑ log τ/ϑ­(*T*_g_/*T*)|_*T*=*T*_g__. It quantifies the deviation of the temperature dependence of the relaxation times from the Arrhenius behavior at *T* > *T*_g_. It can be calculated using the VFT parameters as $m^{*}=\frac{BT_{g}}{2.303{\left(T_{g}-T_{o}\right)}^{2}}$ . A higher (lower) value of fragility corresponds to a steeper increase in the relaxation times near *T*_g_, indicating a more “fragile” (“strong”) glass-forming system. The temperature dependence of the relaxation times for the α and α* processes is shown in Figure 12a in a *T*_g_-scaled plot. The corresponding values of fragility are presented in Figure 12b. Notably, the steepness index increases systematically with increasing molar mass, a trend that is particularly evident for the α process. This observation aligns with experimental results from various homopolymers^41^ and with theoretical predictions,^42,43^ where higher-molar-mass polymers typically exhibit higher fragility. Regarding the effect of secondary structures on the fragility, it can be seen that the slower process (α*), associated with the local relaxation of β-sheets, exhibits consistently lower fragility than the faster process (α), associated with the local relaxation in α-helices, indicating a stronger glass behavior for β-sheets. Both processes originate from amorphous segments located in structurally different environments. The β-sheet secondary structures are stabilized by intermolecular hydrogen bonds, forming a network-like structure. The constraints induced by the latter give rise to a cooperative α* process and to a lower fragility. Conversely, the α-helical secondary structures are stabilized by intramolecular hydrogen bonds. The disordered segments next to the helical segments have more freedom to reconfigure, leading to a more thermally sensitive dynamics. Thus, while both processes originate from disordered segments, it is the structural environment, and, in particular, the extent and type of hydrogen bonding (inter vs intra), that determines their fragility.

**Figure 12:**
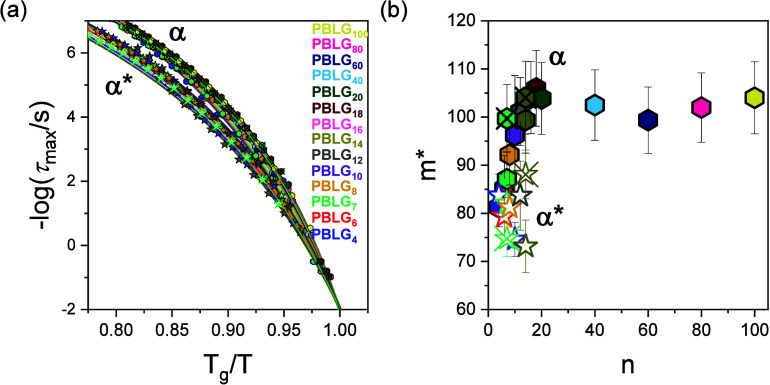
(a) Segmental α and α* relaxations as a function of *T*_g_/*T* for the different degrees of polymerization. (b) Fragility or steepness index, m*, of the two segmental relaxations as a function of molar mass. n-Hexyl-terminated samples are indicated with crossed symbols.

Next, the nature of the α and α* processes, as well as of the slower processes associated with the α-helical and β-sheet secondary structures, was investigated by pressure-dependent measurements (Figure S17). Two representative peptides were selected: PBLG_7(*n*‑hexyl)_, an oligopeptide consisting of a majority of β-sheets, and PBLG_40_, which forms exclusively α-helices.

The pressure dependence of the relaxation times, under isothermal conditions, is presented in Figure 13a,b for the two samples. The *P* dependence of the segmental α and α* processes can be described by the pressure equivalent to the VFT equation as^44^

$$f_{\max}=f_{\infty }\;\exp\left(-\frac{D_{P}P}{P_{0}-P}\right)$$ 5

where τ_ο_ is the segmental relaxation time at atmospheric pressure at a given temperature, *D*_P_ is a dimensionless parameter, and *P*_0_ is the pressure corresponding to the “ideal” glass. In PBLG_7(*n*‑hexyl)_, the segmental (α) process exhibits a stronger pressure dependence than the α* process. In contrast, the slow processes attributed to macrodipole relaxation in the β-sheet and α-helical secondary structures (indicated as β-sheet and as α-helix, respectively) exhibit much weaker pressure dependences.

**Figure 13:**
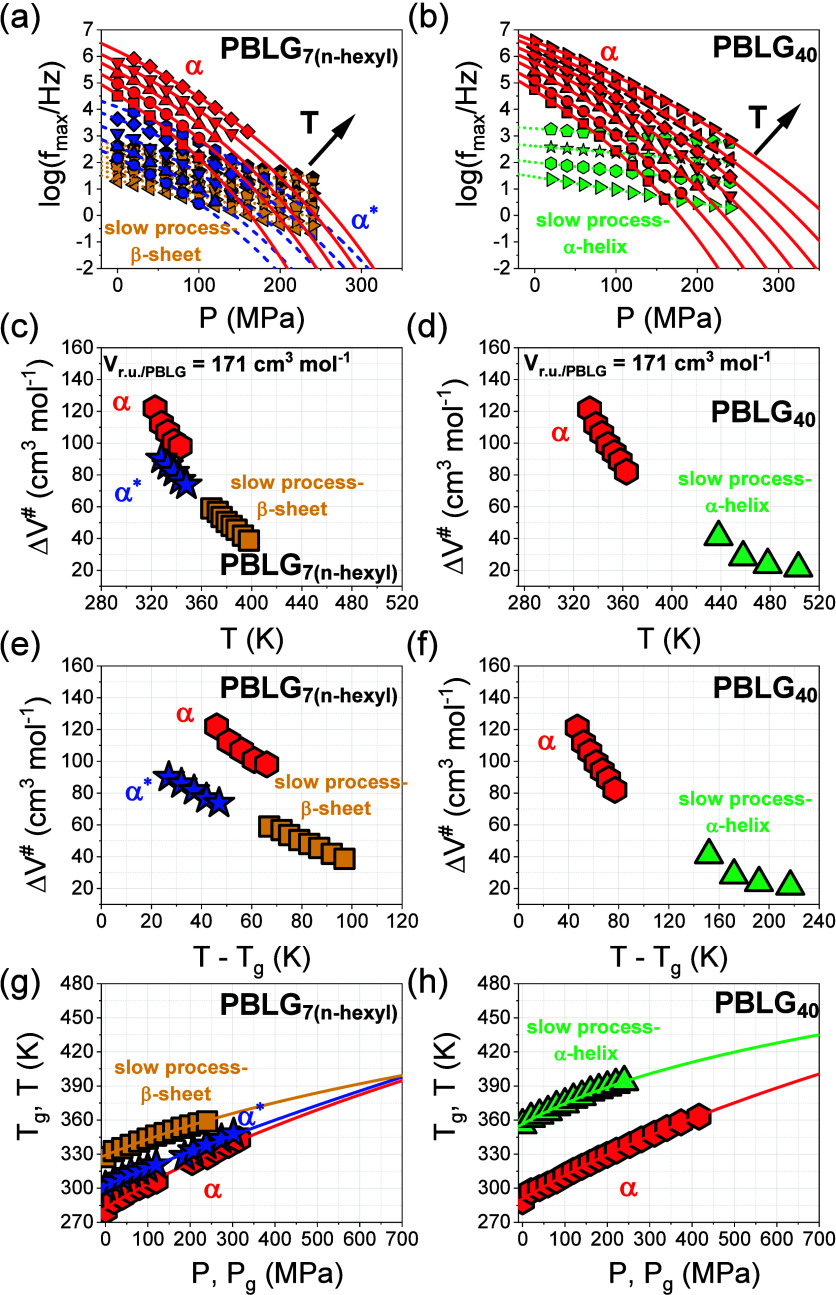
(a) Frequency at a maximum loss as a function of pressure for PBLG_7(*n*‑hexyl)_, corresponding to the segmental (α) process (red) from 323 to 343 K in 5 K steps, the α* process (blue) from 328 to 348 K in 5 K steps, and the β-sheet process (orange) for temperatures of 368, 373, 376, 380, 384, 388, 393, and 398 K. The solid and dashed lines are fits to eq 3, while the dotted lines are fits to an Arrhenius dependence. (b) Pressure dependence of the characteristic frequencies at maximum loss for the PBLG_40_, indicating the segmental (α) process (red) from 333 to 363 K in 5 K steps and the α-helix process (green) for temperatures of 438, 458, 478, and 503 K. The solid and dotted lines are fits to eq 6 and to an Arrhenius dependence, respectively. (c, d) Temperature dependence of the apparent activation volumes (Δ*V* ^#^) for the α (red), α* (blue), β-sheet (orange), and α-helix (green) processes. (e, f) Apparent activation volumes (Δ*V* ^#^) in a *T –* *T*_g_ representation for the respective processes. (g, h) Pressure dependence of the glass temperatures (*T*_g_) as obtained from the isothermal and isobaric representation, corresponding to the freezing of the respective processes, at a characteristic time of τ = 10 s. Solid and dashed lines represent fits to eq 7.

To gain further insight into the pressure-induced behavior of the different relaxation processes, the normalized dielectric strength, Δε/ρ, was examined as a function of pressure. The density was calculated using the Tait equation,^14^ $V\left(P,T\right)=V\left(0,T\right)\{1-0.0894\ln\left(1+\frac{P}{B\left(T\right)}\right)\}$ , where *V*(0, *T*) = *A*_0_ + *A*_1_ *T* + *A*_2_ *T* ^2^ is the perfect volume at atmospheric pressure and *B*(*T*) = *B*_0_ exp­(−*B*_1_ *T*), where *T* is in °C (*A*_0_ = 0.788 cm^3^, *A*_1_ = 4.92 × 10^–4^ cm^3^ °C^–1^, *A*_2_ = 7.57 × 10^–7^ cm^3^ °C^–1^, *B*_0_ = 142 MPa, and *B*_1_ = 4.3 × 10^–3^ °C^–1^). The dielectric strength of a relaxation process is given by^33^

$$\Delta \varepsilon =\frac{1}{3\varepsilon_{o}}gF\frac{\mu^{2}N}{k_{B}TV}$$ 6

where ε_ο_ is the dielectric permittivity of vacuum, *N*/*V* is the number density of dipoles expressed as (ρ/Μ)­Ν_Α_, ρ is the density, *M* is the molar mass of the repeat unit, μ is the dipole moment, *F* is the local field factor, and *g* is the Kirkwood–Fröhlich correlation factor. The normalized dielectric strengths of the aforementioned processes for PBLG_7(*n*‑hexyl)_ and PBLG_40_ are presented in Figure S18. For both the α and α* processes, the normalized dielectric strength, Δε/ρ, for lower pressures increases beyond densification. In the absence of dipole–dipole interactions (g), this implies that pressure induces structural defects (increase in *N* in eq 6) that are incorporated in the segmental processes (i.e., increasing number density of amorphous segments). Following the same reasoning, Δε/ρ for the organized structures should decrease with increasing pressure. This is indeed observed but only for pressures exceeding ∼120 MPa. For *P* < 120 MPa, the normalized dielectric strengths for the α-helix and β-sheet processes also reveal an increase. This could imply dipole–dipole interactions (*g* > 1) of larger entities (i.e., macrodipoles in α-helices).

Pressure-dependent measurements provide access to the apparent activation volume, Δ*V* ^#^.^34,45^ This quantity is extracted from the pressure dependence of the relaxation times (Figure 13a,b), as the slope at each pressure, according to $\Delta V^{\#}=2.303RT{\left(\frac{\partial \log\tau }{\partial P}\right)}_{T}$ . In the absence of hydrogen bonding, Δ*V* ^#^ has been interpreted as reflecting the molecular volume of the underlying dynamic processes. In the presence of h.b., it could associate with a characteristic volume of defects. The apparent activation volume at ambient pressure can be calculated as a function of the temperature for the investigated processes, with the results plotted in Figure 13c,d (and also in Figure 13e,f). For all processes studied, the calculated Δ*V* ^#^ values were found to be smaller than the molar volume of PBLG repeat unit (Δ*V*_r.u._^#^= 171 cm^3^ mol^–1^). However, at *T*_g_, Δ*V* ^#^(α) ≈ Δ*V*_r.u._^#^ and Δ*V* ^#^(α*) < Δ*V*_r.u._^#^. The α process exhibits comparable activation volumes in both PBLG_7(*n*‑hexyl)_ and PBLG_40_, supporting the notion that it originates from the relaxation of amorphous segments in the α-helical peptides. In PBLG_7(*n*‑hexyl)_, the α* process displays a lower activation volume than for the α process at the same temperature but has a similar temperature dependence. This could reflect a smaller defect volume in the case of β-sheets.

Lastly, we examine the pressure sensitivity of the glass temperature, extracted for each process at τ = 10 s (to avoid long extrapolations). Figure 13g,h presents the *P* and *T* dependence of *T*_g_ and *P*_g_, respectively. The data fitted using the empirical equation^34,46^

$$T_{g}\left(P\right)=T_{g}\left(0\right){\left(1+\frac{\nu }{\mu }P\right)}^{1/\nu }$$ 7

with *T*_g_(0) being the glass temperature at atmospheric pressure and μ and ν being fitting parameters. The fitting parameters are shown in Table 3. The pressure sensitivity of *T*_g_ can be discussed with respect to the (d*T*_g_/d*P*)_*P*→0_ coefficient. For amorphous polymers and van der Waals liquids, (d*T*_g_/d*P*)_*P*→0_ is in the range from 360 to 180 K GPa^–1^. On the other hand, hydrogen bonding systems show a weaker dependence, as (d*T*_g_/d*P*)_*P*→0_ ∼ 100 to 20 K GPa^–1^.^34^ The segmental α processes of both PBLG_*7*(m)_ and PBLG_40_ exhibit similar (d*T*_g_/d*P*)_*P*→0_ parameters (∼200 K GPa^–1^), consistent with the values reported for α-helical polypeptides (PBLG and PZLL).^14^ In contrast, the segmental α* process of PBLG_7(*n*‑hexyl)_ exhibits a lower pressure coefficient of 158 K GPa^–1^, closely related to the behavior observed for the β-sheet-forming PGly.^14^ These findings again highlight the role of the secondary structure in governing pressure sensitivity. The network-like structure of β-sheets imposes stronger constraints on the amorphous segments, thereby limiting the pressure response of the segmental α* process.

**Table 3:** Parameters of the *T*_g_ (*P*) Dependence (Equation 7)

process	ν	μ (MPa)	(**d*T*_g_**/**d*P***)_*P→* **0**_ **(K GPa** ^**–1**^ **)**
segmental α process (PBLG_7(*n*‑hexyl)_)	3.1 ± 0.9	1210 ± 150	234
segmental α* process (PBLG_7(*n*‑hexyl)_)	2.2 ± 0.8	1910 ± 80	158
“slow” β-sheet process (PBLG_7(*n*‑hexyl)_)	6.5 ± 1.1	1705 ± 130	208
segmental α process (PBLG_40_)	2.9 ± 0.3	1320 ± 60	220
“slow” α-helix process (PBLG_40_)	5.1 ± 1.3	2151 ± 140	153

So far, the local dynamics has revealed two distinct glass temperatures in polypeptides that exhibit both secondary structures, each corresponding to the relaxation of amorphous segments interrupting α-helices and β-sheets. ^13^C NMR and DS showed that β-sheets relax at longer timescales, indicating a more cooperative relaxation. The global dynamics of the α-helical and β-sheet macrodipoles were also evident at even longer timescales. Further analysis revealed consistently lower fragility values for β-sheets. This behavior was discussed in view of the constraints imposed by their inherent network-like structure stabilized by intermolecular hydrogen bonds. In contrast, α-helices (stabilized by intramolecular h.b.) allow for greater mobility in nearby amorphous segments, leading to a more thermally sensitive dynamics. These structural differences were also reflected in the lower pressure coefficient of β-sheets compared to α-helices. Given the important differences found for β-sheets, it would be interesting to generalize the findings by studying intrinsically β-sheet-forming polypeptides at higher molar masses, e.g., by synthetic methods explored recently for polypeptides and their block copolymers.^47^

#### 3.3.3 Viscoelastic Response

Here, we examine the viscoelastic properties of PBLG as a function of molar mass and chain-end-group chemistry. Figure 14 depicts master curves of the storage (*G*′) and the loss (*G″*) moduli as a function of frequency. For both oligopeptides and polypeptides, the storage modulus consistently exceeds the loss modulus (*G*′ > *G″*), indicating a solid-like (elastic) behavior. The solid-like response indicates the underlying structural organization, i.e., the α-helical and β-sheet secondary structures and their supramolecular self-assembly into ordered domains. The respective van Gurp–Palmen (vGP) plot of the phase angle (δ) versus the complex modulus (|G*|), shown in Figure 15, is more informative of the applicability of time–temperature superposition (*tT*s) and of the elastic plateau. For homopolymers following the *tT*s, all isothermal data in a vGP plot overlap in a single master curve. In this case, there exist two minima in δ. One at the plateau modulus $G_{N}^{0}$ , reflecting the elastic properties of the entanglement “network” (typically $G_{N}^{0}$ ∼ 10^5^ Pa) and another in the vicinity of the liquid-to-glass temperature (*T*_g_) where |G*| ∼ 10^9^ Pa. Between the two minima, δ increases and goes through a maximum.

**Figure 14:**
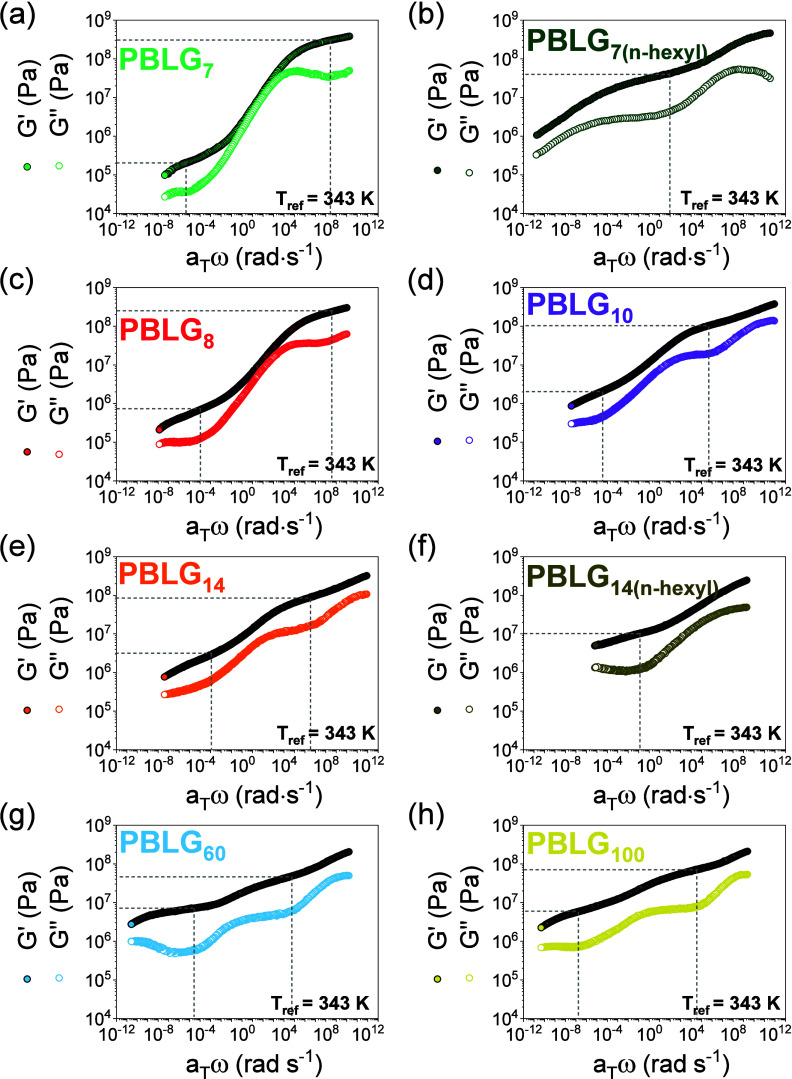
Master curves of the storage (filled circles) and loss (open circles) moduli of PBLG_7_ (green), PBLG_7(*n*‑hexyl)_ (dark green), PBLG_8_ (red), PBLG_10_ (purple), PBLG_14_ (orange), PBLG_14(*n*‑hexyl)_ (dark yellow), PBLG_60_ (blue), and PBLG_100_ (yellow). There is a solid-like behavior (*G′* > *G″*) across all measured frequencies.

**Figure 15:**
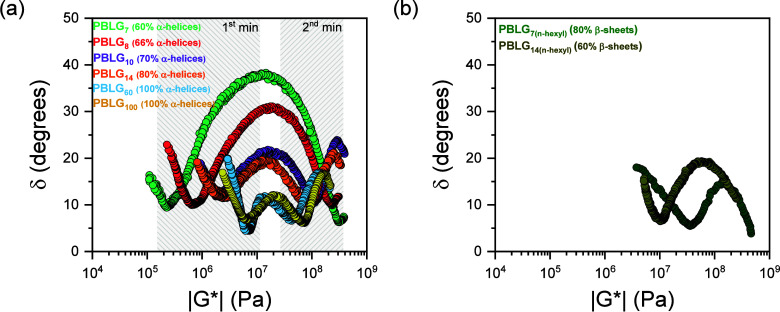
van Gurp–Palmen plot for the investigated (a) dimethylamino-terminated and (b) *n*-hexyl-terminated samples with respective majority of α-helices and β-sheets. Colors indicate the different degrees of polymerization: PBLG_7_ (green), PBLG_7(*n*‑hexyl)_ (dark green), PBLG_8_ (red), PBLG_10_ (purple), PBLG_14_ (orange), PBLG_14(*n*‑hexyl)_ (dark yellow), PBLG_60_ (blue), and PBLG_100_ (yellow). In the former samples, two minima are evident, while in the latter, a single minimum is shown.

In the dimethylamino-terminated samples, the vGP plot (Figure 15a) reveals two distinct minima. In the case of PBLG_7_ (60% of α-helices), the first δ_min_ is at a modulus of |G*| ∼ 2 × 10^5^ Pa, reminiscent of amorphous polymers. However, increasing molar mass brings about a large shift toward higher |G*| values, from 2 × 10^5^ Pa in PBLG_7_ to 7 × 10^6^ Pa in PBLG_100_. Naturally, we assign this first δ_min_ to the α-helical segments that grow in number and coherence with *n* (Figure 4). For polypeptides, where the hierarchical self-assembly (α-helices and their hexagonal packing) is more coherent, δ_min_ ∼ 4° and the plateau becomes more evident. The significant increase in the |G*| values with *n* suggests a percolation mechanism of the helical structures. To quantify the length scale of these structural constraints, we calculate an apparent molar mass, *M*_e_, and the corresponding *N*_e_, of the smallest structural unit that imparts rigidity using the equation $G_{N}^{0}=\frac{4}{5}\frac{\rho RT_{\mathrm{ref}}}{M_{e}}$ ,^48^ where ρ is the density at the reference temperature (*T*_ref_ = 343 K). *N*_e_ decreases with increasing *n* (Figure S19), ranging from about 60 repeat units in PBLG_7_ to only 2 repeat units for PBLG_100_, reflecting increasing topological constraints in the α-helical polypeptides with a decreasing “mesh” size. As an additional approach, the characteristic length of these constraints (i.e., the “mesh” size), *d*, can also be estimated using $G_{N}^{0}=\frac{k_{B}T_{\mathrm{ref}}}{d^{3}{\left(\omega_{c}\tau \right)}^{2}}$ , where *k*_B_ is the Boltzmann constant, *T*_ref_ is the superposition reference temperature, and ω_c_τ = 1. The results are shown in the inset to Figure S19. The consistency between the two approaches (Figure S19) reinforces the idea that increased chain length promotes tighter coupling between the helical segments, leading to a more rigid “network”. This reflects the critical role of hierarchical ordering, from individual α-helices to their collective packing, in the viscoelastic properties of the system. In the case of the *n*-hexyl-terminated samples (Figure 15b), the first minimum in the vGP plot corresponds to high |G*| values. This high-modulus elastic plateau is associated with the compact, network-like structures of the β-sheets. We can also observe that well-ordered α-helices (in PBLG_60_ and PBLG_100_) and β-sheets (in PBLG_7(*n*‑hexyl)_ and PBLG_14(*n*‑hexyl)_) exhibit similar plateau values (∼10^7^ Pa), reflecting increasing topological constraints through percolation/aggregation. Hence, rheological measurements reveal the emergence of a supramolecular organization, an interconnected “mesh” of ordered domains, whose formation is driven by percolation/aggregation of secondary motifs.

At high |G*| (∼10^9^ Pa), a second minimum is evident, suggesting proximity to the liquid-to-glass “transition” regime. To verify its origin and possibly associate this feature with the two segmental processes (α and α*), we extracted the relaxation times from *G″*(ω) at low temperatures and compared it with the dynamics as seen in DS (Figure S20). There is an excellent agreement of the extracted timescales with the ones corresponding to the two processes in DS. For low and intermediate degrees of polymerization, the second, well-resolved δ_min_ reflects the freezing of the segments participating in the α* process in DS. In polypeptides, the extracted relaxation times align with the weak intermediate process (indicated as I in Figure S20). Moreover, there is evidence for a second process at even higher frequencies associated with the α-process in the α-helical PBLGs.

In conclusion, polypeptides because of their extensive self-organization exhibit an elastic response (*G′* > *G″*). The different secondary structures have a clear viscoelastic signature at the segmental level and a similar fingerprint at the domain level, with a decreasing “mesh” size by increasing molar mass. In addition, the high moduli of the oligopeptides with the β-sheet motifs make them ideal candidates as load-bearing scaffolds or implantable supports. On the other hand, α-helical polypeptides with low molar masses are suitable for applications involving compliant tissues such as the epithelium, basement membranes, soft connective tissues, or even retinal substrates.^23,24^

## 4 Conclusions

A series of poly­(γ-benzyl-l-glutamate) (PBLG) peptides were synthesized, with a broad range of molar masses: from oligopeptides with a high β-sheet content to polypeptides exclusively forming α-helical structures. By combining static (SAXS and ^13^C solid-state NMR) with dynamic probes (^13^C solid-state NMR, DS as a function of temperature and pressure, and rheology), we identified distinct local and global dynamics of α-helices and β-sheets.

In oligopeptides, two glass temperatures (*T*_g_s) were identified by DSC and DS that exhibit both secondary structures: the lower/higher *T*_g_ associated with the segmental relaxations of amorphous segments interrupting the α-helices/β-sheets and at their chain-ends. The higher *T*_g_ associated with the segmental relaxation of β-sheets indicates significantly more cooperative dynamics. This is the first report for β-sheet-associated *T*_g_ in a completely nonhydrated polypeptide. The relaxation of the α-helical and β-sheet macrodipoles was also evident at longer timescales. Again, it is the first time that β-sheets are shown to have chain dynamics associated with dipoles perpendicular to the chain. The differences between the two secondary structures were also evident in the fragility or steepness index and their pressure dependence. β-Sheets, stabilized by intermolecular hydrogen bonds, exhibited slower dynamics, a lower fragility, and a weaker pressure response. In contrast, α-helices, organized through intramolecular hydrogen bonds, showed a faster segmental process, a more fragile behavior, and a higher pressure sensitivity. Evidently, it is the type of hydrogen bond that determines the dynamic behavior.

The viscoelastic results revealed that both α-helices and β-sheets form distinct superstructures, as evidenced by their different viscoelastic signatures. Expectedly, due to their self-organization, an elastic response (*G′* > *G″*) was observed across all samples. The van Gurp–Palmen (vGP) plot analysis revealed correlations between the elastic modulus and the dominant secondary structure. Two minima in the phase angle, δ, were identified: one at the plateau modulus $G_{N}^{0}$ reflecting the elastic properties of the entanglement “network” (∼10^5^ Pa) and another in the vicinity of the *T*_g_ (∼10^9^ Pa). In low-molar-mass α-helix-rich peptides, we observed molar-mass-dependent mesh sizes, yielding compliant matrices with moduli spanning the range of soft biological tissues (1–20 kPa for epithelial tissues, 4–10 kPa for basement membranes). Conversely, β-sheet-rich peptides displayed compact, network structures with significantly higher moduli ( $G_{N}^{0}$ ∼ 10^7^ Pa). Their mechanical properties approach those of load-bearing biological tissues (e.g., tendon at ∼1 GPa) and synthetic bone substitutes (15–20 GPa range). This supramolecular level of organization emerged through the percolation/aggregation of secondary structural motifs.

Overall, our results demonstrate that chain length and end-group chemistry can be used to selectively stabilize α-helices and/or β-sheets, enabling deliberate control over the structural, dynamical, and viscoelastic properties of polypeptides. These findings possibly provide a framework for designing polypeptides tailored to specific biomedical applications: from rigid, load-bearing scaffolds, implantable supports, and drug delivery matrices (harnessing β-sheet rigidity) to soft tissue engineering platforms, such as cell–support matrices and biointerfaces (exploiting the tunability of α-helical networks). Of course, for such applications, additional mechanical properties (e.g., toughness, brittleness, particularly for low degrees of polymerization, fatigue resistance, and viscoelasticity/strain rate dependence) and processing/biological considerations would need to be engineered.

## Supplementary Material



## Notes

Materials, analytical methods, and physical properties (DSC, WAXS, solid-state NMR, dielectric spectroscopy, and viscoelasticity) (PDF)

## References

[1] Branden, C. ; Tooze, J. Introduction to Protein Structure; Garland: New York, 1991.

[2] Huang P. S., Boyken S. E., Baker D. (2016). The Coming of Age of De Novo Protein Design. Nature.

[3] Doster W., Cusack S., Petry W. (1989). Dynamical Transition of Myoglobin Revealed by Inelastic Neutron Scattering. Nature.

[4] Schmidt M., Achterhold K., Prusakov V., Parak F. G. (2009). Protein Dynamics of a β-sheet Protein. Eur. Biophys. J..

[5] Vitkup D., Ringe D., Petsko G. A., Karplus M. (2000). Solvent Mobility and the Protein ‘Glass’ Transition. Nat. Struct. Biol..

[6] Karplus M., McCammon J. A. (2002). Molecular Dynamics Simulations of Biomolecules. Nat. Struct. Biol..

[7] Iben I. E. T., Braunstein D., Doster W., Frauenfelder H., Hong M. K., Johnson J. B., Luck S., Ormos P., Schulte A., Steinbach P. J., Xie A. H., Young R. D. (1989). Glassy Behavior of a Protein. Phys. Rev. Lett..

[8] Fenimore P. W., Frauenfelder H., McMahon B. H., Parak F. G. (2002). Slaving: Solvent Fluctuations Dominate Protein Dynamics and Functions. Proc. Natl. Acad. Sci. U.S.A..

[9] Fenimore P. W., Frauenfelder H., McMahon B. H., Young R. D. (2004). Bulk-Solvent and Hydration-Shell Fluctuations, Similar to α - and β -Fluctuations in Glasses, Control Protein Motions and Functions. Proc. Natl. Acad. Sci. U.S.A..

[10] Swenson J., Jansson H., Bergman R. (2006). Relaxation Processes in Supercooled Confined Water and Implications for Protein Dynamics. Phys. Rev. Lett..

[11] Jansson H., Bergman R., Swenson J. (2011). Role of Solvent for the Dynamics and the Glass Transition of Proteins. J. Phys. Chem. B.

[12] Cerveny S., Combarro-Palacios I., Swenson J. (2016). Evidence of Coupling between the Motions of Water and Peptides. J. Phys. Chem. Lett..

[13] Cerveny S., Swenson J. (2019). Water Dynamics in the Hydration Shells of Biological and Non-Biological Polymers. J. Chem. Phys..

[14] Papadopoulos P., Floudas G., Schnell I., Klok H. A., Aliferis T., Iatrou H., Hadjichristidis N. (2005). “Glass Transition” in Peptides: Temperature and Pressure Effects. J. Chem. Phys..

[15] Floudas G., Spiess H. W. (2009). Self-Assembly and Dynamics of Polypeptides. Macromol. Rapid Commun..

[16] Papadopoulos P., Floudas G., Klok H.-A., Schnell I., Pakula T. (2004). Self-Assembly and Dynamics of Poly­(γ-benzyl-L-glutamate) Peptides. Biomacromolecules.

[17] Magoshi J., Nakamura S. (1975). Studies on Physical Properties and Structure of Silk. Glass Transition and Crystallization of Silk Fibroin. J. Appl. Polym. Sci..

[18] Agarwal N., Hoagland D. A., Farris R. J. (1997). Effect of Moisture Absorption on the Thermal Properties of Bombyx Mori Silk Fibroin Films. J. Appl. Polym. Sci..

[19] Hu X., Kaplan D., Cebe P. (2006). Determining Beta-sheet Crystallinity in Fibrous Proteins by Thermal Analysis and Infrared Spectroscopy. Macromolecules.

[20] Hu X., Kaplan D., Cebe P. (2008). Dynamic Protein-Water Relationships During β-sheet Formation. Macromolecules.

[21] Yu L., Hu X., Kaplan D., Cebe P. (2010). Dielectric Relaxation Spectroscopy of Hydrated and Dehydrated Silk Fibroin Cast from Aqueous Solution. Biomacromolecules.

[22] Mieda S., Aida M. (2013). Macrodipole Moment of Polypeptides in β-Sheet and Its Prediction from Dipole Moments of Amino Acid Residues as Building Blocks: Alanine and Glycine in β-Strand. Chem. Lett..

[23] Wagner, W. R. ; Sakiyama-Elbert, S. E. ; Zhang, G. ; Yaszemski, M. J. ″Biomaterials Science: An Introduction to Materials in Medicine″, Academic Press, Elsevier, 4th Ed., San Diego, US, 2020, 1–1586.

[24] Thomasy S. M., Raghunathan V. K., Winkler M., Reilly C. M., Sadeli A. R., Russell T. P., Jester J. V., Murphy C. J. (2014). Elastic Modulus and Collagen Organization of the Rabbit Cornea: Epithelium to Endothelium. Acta Biomater..

[25] Mavrogiorgis D., Bilalis P., Karatzas A., Skoulas D., Fotinogiannopoulou G., Iatrou H. (2014). Controlled Polymerization of Histidine and Synthesis of Well-defined Stimuli Responsive Polymers. Elucidation of the Structure-Aggregation Relationship of this Highly Multifunctional Material. Polym. Chem..

[26] Vinod Chandran C., Madhu P. K., Kurur N. D., Bräuniger T. (2008). Swept-Frequency Two-Pulse Phase Modulation (SWf-TPPM) Sequences with Linear Sweep Profile for Heteronuclear Decoupling in Solid-State NMR. Magn. Reson. Chem..

[27] Guan X., Stark R. E. (2010). A General Protocol for Temperature Calibration of MAS NMR Probes at Arbitrary Spinning Speeds. Solid State Nucl. Magn. Reson..

[28] Shoji A., Ozaki T., Saito H., Tabeta R., Ando I. (1984). Conformational Characterization of Solid Polypeptides by 13C NMR Recorded by the Cross Polarization-Magic Angle Spinning Method: Conformation-Dependent 13C Chemical Shifts of Oligo-and Poly­(γ-benzyl L-glutamates) and Sequential Copolymers of γ-Benzyl and γ-Methyl L-Glutamates and Qualitative Evaluation of Side-Chain Orientation. Macromolecules.

[29] Massiot D., Fayon F., Capron M., King I., Lecalve S., Alonso B., Durand J. O., Bujoli B., Gan Z., Hoatson G. (2002). Modelling One-and Two-Dimensional Solid-State NMR Spectra. Magn. Reson. Chem..

[30] Saalwächter K., Schnell I. (2002). REDOR-Based Heteronuclear Dipolar Correlation Experiments in Multi-Spin Systems: Rotor-Encoding, Directing, and Multiple Distance and Angle Determination Solid State Nucl. Magn. Reson..

[31] Saalwächter K., Spiess H. W. (2001). Heteronuclear 1H–13C Multiple-Spin Correlation in Solid-State Nuclear Magnetic Resonance: Combining Rotational-Echo Double-Resonance Recoupling and Multiple-Quantum Spectroscopy. J. Chem. Phys..

[32] Langer B., Schnell I., Spiess H. W., Grimmer A.-R. (1999). Temperature Calibration under Ultrafast MAS Conditions. J. Magn. Reson..

[33] Kremer, F. ; Schönhals, A. in Broadband Dielectric Spectroscopy; Springer Berlin, Heidelberg, 2002, 1–729.

[34] Floudas, G. ; Paluch, M. ; Grzybowski, A. ; Ngai, K. L. in Molecular Dynamics of Glass-Forming Systems. Effects of Pressure; Springer-Verlag Berlin Heidelberg, 2011, 1–174.

[35] Wubbenhorst M., van Turnhout J. (2002). Analysis of Complex Dielectric Spectra. I. One-Dimensional Derivative Techniques and Three-Dimensional Modelling. J. Non-Cryst. Solids.

[36] Floudas G., Papadopoulos P., Klok H. A., Vandermeulen G. W. M., Rodriguez-Hernandez J. (2003). Hierarchical Self-Assembly of Poly­(γ-benzyl-l-glutamate)-Poly­(ethylene glycol)-Poly­(γ-benzyl-l-glutamate) Rod-Coil-Rod Triblock Copolymers. Macromolecules.

[37] Southern S. A., Perras F. A. (2024). Comparison of Methods for the NMR Measurement of Motionally Averaged Dipolar Couplings. J. Magn. Reson..

[38] Stockmayer W. H. (1967). Dielectric Dispersion in Solutions of Flexible Polymers. Pure Appl. Chem..

[39] Papamokos G., Wudarczyk J., Graf R., Schollmeyer D., Baumgarten M., Müllen K., Floudas G. (2018). Dipolar Relaxation in Functionalized Poly-p-phenylenes Bearing Ultra-Strong Dipoles Perpendicular to the Backbone. Macromolecules.

[40] Ngai K. L., Roland C. M. (1993). Chemical Structure and Intermolecular Cooperativity: Dielectric Relaxation Results. Macromolecules.

[41] Kunal K., Robertson C. G., Pawlus S., Hahn S. F., Sokolov A. P. (2008). Role of Chemical Structure in Fragility of Polymers: A Qualitative Picture. Macromolecules.

[42] Dudowicz J., Freed K. F., Douglas J. F. (2005). Fragility of Glass-Forming Polymer Liquids. J. Phys. Chem. B.

[43] Saltzman E. J., Schweizer K. S. (2007). Short Time Properties, Dynamic Fragility and Pressure Effects in Deeply Supercooled Polymer Melts. J. Phys.: Condens. Matter.

[44] Paluch M., Patkowski A., Fischer E. W. (2000). Temperature and Pressure Scaling of the α Relaxation Process in Fragile Glass Formers: A Dynamic Light Scattering Study. Phys. Rev. Lett..

[45] Mpoukouvalas K., Floudas G., Williams G. (2009). Origin of the α, β, (βα), and ″Slow″ Dielectric Processes in Poly­(ethyl methacrylate). Macromolecules.

[46] Andersson S. P., Andersson O. (1998). Relaxation Studies of Poly­(propylene glycol) under High Pressure. Macromolecules.

[47] Kambale P., Nisal R., Jayakannan M. (2025). Synthetic Strategy to Build High-Molecular-Weight Poly­(L-tyrosine) and Its Unexplored β-Sheet Block Copolymer Nanoarchitectures. Biomacromolecules.

[48] Fetters L. J., Lohse D. J., Graessley W. W. (1999). Chain Dimensions and Entanglement Spacings in Dense Macromolecular Systems. Polym. Sci. Part B: Polym. Phys..

