# Supplementary material for: Distinct Local and Global Dynamics of α‑Helices and β‑Sheets in Poly(γ-benzyl‑l‑glutamate) Peptides
Source: Biomacromolecules. 2025 Dec 19;27(1):855–72. doi: 10.1021/acs.biomac.5c02128 (PMC12806854; doi:10.1021/acs.biomac.5c02128)
Supplement: Supplementary file 1 [file bm5c02128_si_001.pdf]

SUPPORTING INFORMATION

Distinct Local and Global Dynamics of α-helices and β-sheets in Poly(γ-benzyl-L-glutamate) Peptides

Marianna Spyridakou,[1] Iren G. Stavrakaki,[2] Evangelia Tsagkaraki,[2] Christina Varfi,[2] Robert Graf,[3] Hermis Iatrou,[2 *] George Floudas[1,3,4 *]

[1] *Department of Physics, University of Ioannina, P.O. Box 1186, 451 10 Ioannina, Greece*

[2] *Department of Chemistry, University of Athens, 15771 Athens, Greece*

[3] *Max Planck Institute for Polymer Research, Ackermannweg 10, 55128 Mainz, Germany*

[4] *University Research Center of Ioannina (URCI) - Institute of Materials Science and Computing, 45110 Ioannina, Greece*

***Corresponding Aurhors:** Hermis Iatrou: iatrou@chem.uoa.gr, George Floudas: gfloudas@uoi.gr

Contents

I. METHODS AND MATERIALS

Materials

Analytical Methods

a. Proton Nuclear Magnetic Resonance Spectroscopy

b. Size Exclusion Chromatography

c. Fourier Transform Infrared

Synthesis and characterization of *γ*-benzyl-L-glutamate N-carboxy anhydride (BLG-NCA)

Synthesis of poly(*γ*-benzyl-L-glutamate) (PBLG)

II. PHYSICAL PROPERTIES

a. Thermal properties

b. Wide-angle X-ray scattering

c. Solid state NMR

d. Dielectric spectroscopy

e. Viscoelasticity

III. REFERENCES

I. METHODS AND MATERIALS

Materials

H-Glu(OBzl)-OH was purchased from Bachem AG. Limonene (97%) and diethyl ether (≥99%) were acquired from Aldrich, while ethyl acetate (EtOAc, >99.5%) underwent fractional distillation over phosphorus pentoxide, and n-hexane (>99%) was purchased from Merck Millipore and was dried over n-butyl lithium. *N*,*N*-Dimethylformamide (DMF) (Fischer Scientific, 99.9+%, special grade for peptide synthesis with less than 50 ppm of active impurities), the polymerization solvent, was further purified by short-path fractional distillation under high vacuum in a custom-made apparatus, with only the middle fraction being used. Triphosgene (99%) was purchased from Acros Organics. All other solvents and reagents were purchased from commercial suppliers and used as received.

Analytical Methods

a. Proton Nuclear Magnetic Resonance Spectroscopy (^{1}H-NMR) (400 MHz) was conducted on a Bruker 400 spectrometer. The spectra of the polymeric materials as well as the monomers (N-carboxy anhydrides (NCAs)) were recorded in $CDCl_3$, at ambient temperature using Bruker's standard pulse programs for proton ^{1}H and COSY.

b. Size Exclusion Chromatography (SEC) analysis was performed using two SEC sets. One was composed of a Waters Breeze instrument (Milford, MA, USA) equipped with a 2410 differential refractometer and a Precision PD 2020 two angles (15°, 90°) light scattering detector (TALLS). The carrier solvent was 0.05% potassium trifluoroacetate (KTFA) solution of HFIP at a flow rate of 1 mL min^{-1} at 40 ºC. A PSS PFG precolumn, a PSS PFG 100 Å, and a PSS PFG 1000 Å columns placed in series were employed. The concentration that was utilized was 1 mg mL^{-1} and sonication was performed when needed. A second SEC instrument was used for the analysis of the polypeptides. The system was composed of a Waters 600 HPLC pump, Waters Ultrastyragel columns (HT-2, HT-4), a Waters 410 differential refractometer, and a Precision PD 2020 two angles (15°, 90°) light scattering detector (TALLS) operating at 60 °C. The carrier solvent used was a solution of 0.1 M LiBr in DMF with a flow rate of 1 mL min^{-1}. The concentration measured was 6 mg mL^{-1} and sonication was performed when needed.

c. Fourier Transform–Infrared (FT-IR) measurements were performed with a Perkin Elmer Spectrum One instrument, recording in KBr pellets at room temperature across a range of 450–4000 cm^{-1}.

Synthesis and characterization of *γ*-benzyl-L-glutamate N-carboxy anhydride (BLG-NCA)

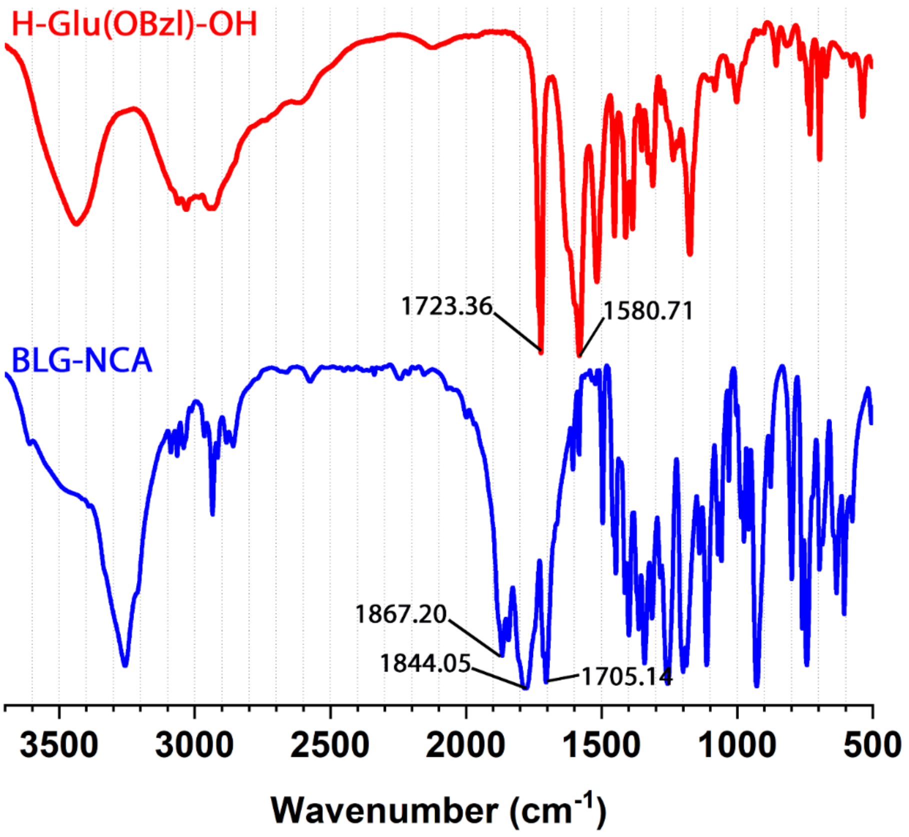

Figure S1. FT-IR spectra: (red) of H-Glu(OBzl)-OH precursor compound, (blue) final BLG-NCA product after recrystallizations.

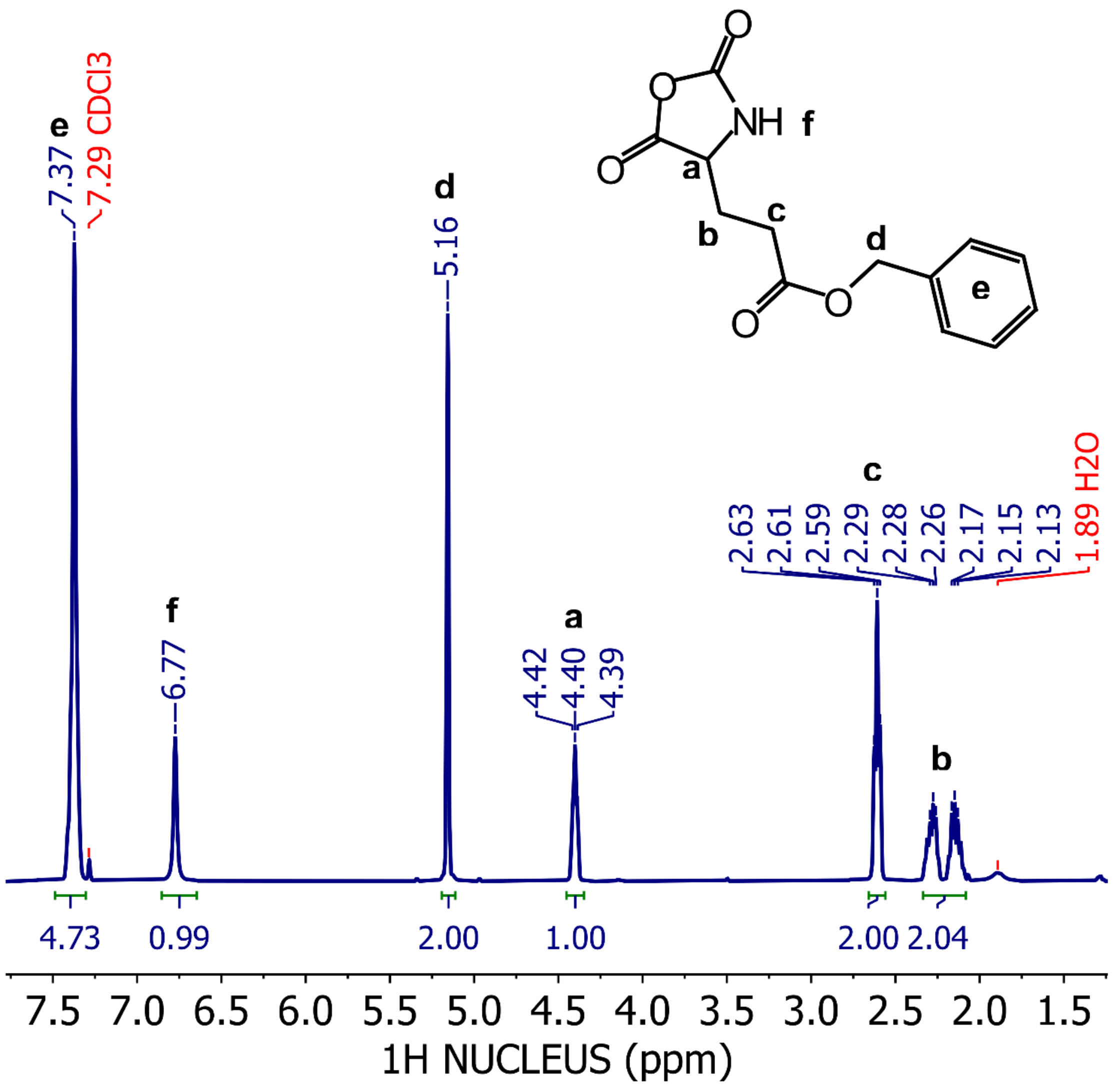

Figure S2. ^{1}H-NMR spectrum of BLG-NCA in $CDCl_3$.

Additionally, the purity of γ-benzyl-L-glutamate NCA was confirmed by ^{1}H-NMR in $CDCl_3$ (Figure 2). The observed peaks corresponded to the NCA hydrogens with integration values matching the theoretical ones. ^{1}H-NMR (400 MHz, $CDCl_3$, δ, ppm): (b) appears at 2.13-2.29 ppm (2H, -CH-CH_2-CH_2-CO-), (c) at 2.58-2.61 ppm (2H, -CH-CH_2-CH_2-CO-), (a) at 4.39-4.42 ppm (1H, N-CH-CO), (d) at 5.16 ppm (2H, Phenyl-CH_2-O-CO-), (f) at 6.77 ppm (1H, - CH_2-NH-CO- of the anhydride) and (e) at 7.37 ppm (5H of phenyl group).

Synthesis of poly(γ-benzyl-L-glutamate) (PBLG)

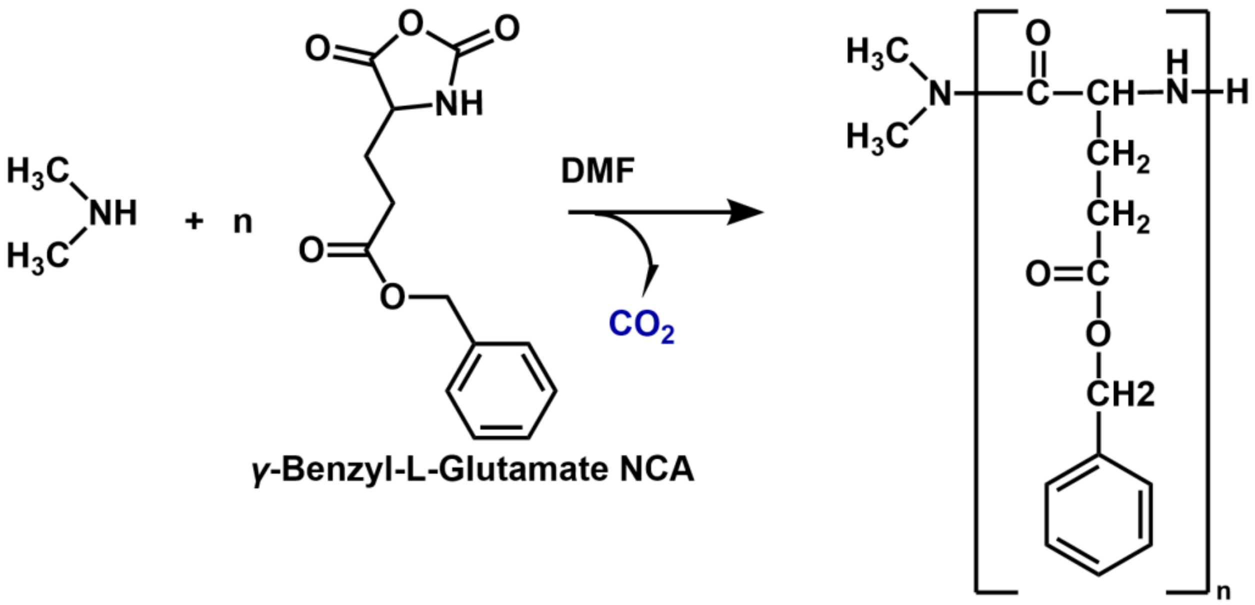

Scheme S1. Reaction used for the synthesis of PBLG polypeptides.

Characterization of the series of PBLG polypeptides

A. Fourier-transform infrared (FT-IR) spectroscopy

Fourier-transform infrared (FT-IR) spectroscopy was used to verify the successful completion of the polymerization process. As illustrated in Figure 3, the disappearance of characteristic NCA peaks (at 1867 cm^{-1} and 1844 cm^{-1}), along with the emergence of a new absorption band around 1650 cm^{-1}(highlighted in the left green area), corresponding to the amide bond, supports this conclusion. Additionally, a distinct peak observed at approximately 1730 cm^{-1}(left green area) indicates the presence of the benzyl ester group, while signals in the 600–800 cm^{-1} range (right green area) are attributed to the vibrations of the aromatic ring, confirming the retention of the protective group, throughout the polymerization process

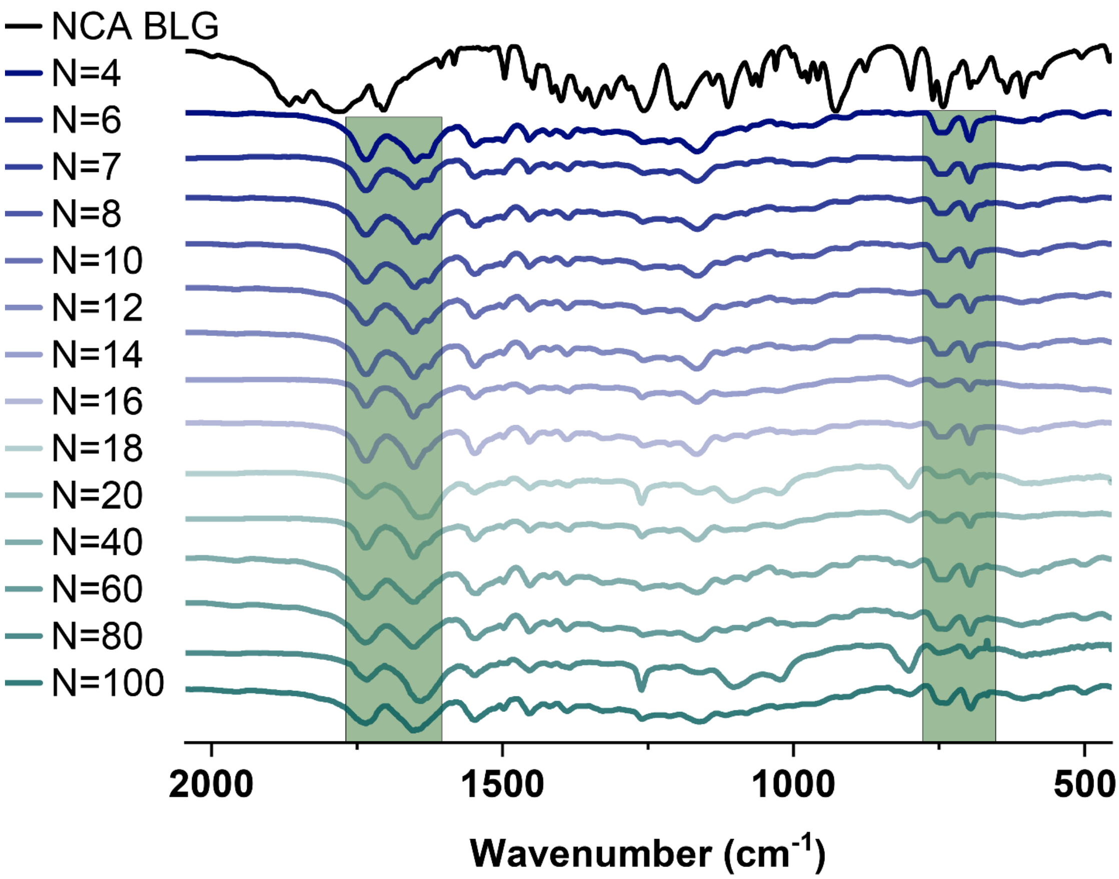

Figure S3. FT-IR spectra of BLG-NCA compared to every homopolymer, starting from 4 monomeric units up to 100.

B. NMR spectroscopy.

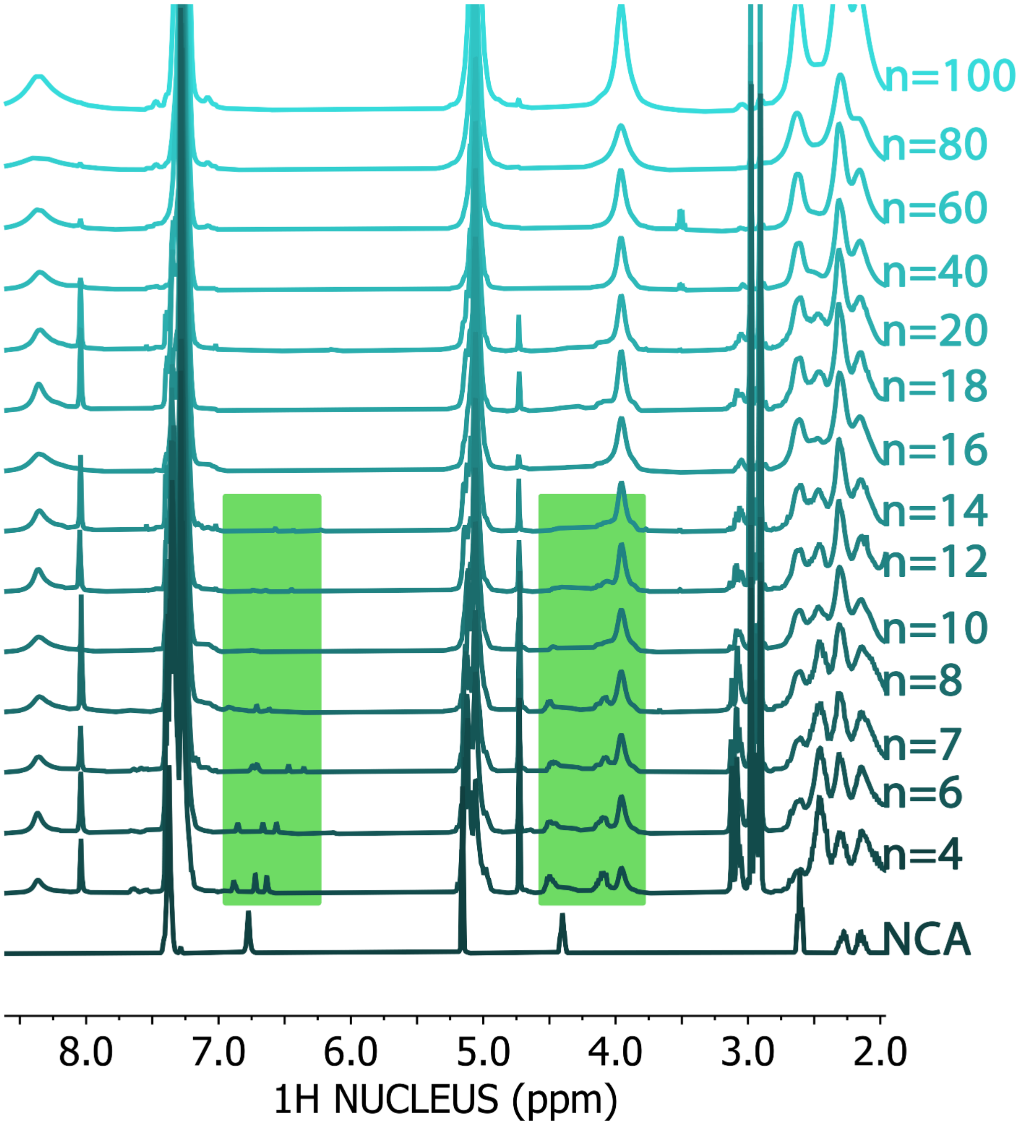

Figure S4. NMR spectra of BLG-NCA compared to every homopolymer, starting from 4 monomeric units up to 100.

The ^{1}H-NMR spectra of the homopolymers are presented in Figure S4. **n= 4-** ^{1}H NMR (400 MHz, $CHCl_3$, δ in ppm): (b,c) 2.05–2.73 (4H, CH2–CH2–C=O benzyl ester), (DMA) 2.88-3.14 (6H, $((CH_3)_2)$, (a) 3.80-4.54 (1H, (C=O)-C-H-N),(d) 4.90-5.19 (2H, (C=O)-O-CH2 benzene), (e) 6.63-7.49 (5H, =C-H aromatic), (i) 8.19-8.50 (1H, –NH– of the main polypeptide chain) **n= 6-** ^{1}H NMR (400 MHz, $CHCl_3$, δ in ppm): (b,c) 2.05–2.67 (4H, CH2–CH2–C=O benzyl ester), (DMA) 2.84-3.17 (6H, $((CH_3)_2)$,(a) 3.77-4.55 (1H, (C=O)-C-H-N), (d) 4.95-5.19 (2H, (C=O)-O-CH2 benzene), (e) 6.52-7.45 (5H, =C-H aromatic), (i) 8.15-8.57 (1H, –NH– of the main polypeptide chain) **n= 7-** ^{1}H NMR (400 MHz, $CHCl_3$, δ in ppm): (b,c) 2.13–2.65 (4H, CH2–CH2–C=O benzyl ester), (DMA) 2.05-3.15 (6H,

(($(CH_3)_2$), (a) 3.80-4.52 (1H, (C=O)-C-H-N), (d) 4.91-5.19 (2H, (C=O)-O-CH2 benzene), (e) 6.35-7.46 (5H, =C-H aromatic), (i) 8.12-8.50 (1H, –NH– of the main polypeptide chain) **n= 8**- ^{1}H NMR (400 MHz, $CHCl_3$, δ in ppm): (b,c) 1.90–2.73 (4H, CH2–CH2–C=O benzyl ester), (DMA) 2.90-3.16 (6H, (($CH_3)_2$), (a) 3.82-4.57 (1H, (C=O)-C-H-N),(d) 4.91-5.20 (2H, (C=O)-O-CH2 benzene), (e) 6.57-7.46 (5H, =C-H aromatic), (i) 8.17-8.47 (1H, –NH– of the main polypeptide chain) **n= 10**- ^{1}H NMR (400 MHz, $CHCl_3$, δ in ppm): (b,c) 2.01–2.68 (4H, CH2–CH2–C=O benzyl ester), (DMA) 2.87-3.19 (6H, (($CH_3)_2$), (a) 3.80-4.53 (1H, (C=O)-C-H-N),(d) 4.94-5.23 (2H, (C=O)-O-CH2 benzene), (e) 6.71-7.47 (5H, =C-H aromatic), (i) 8.11-8.54 (1H, –NH– of the main polypeptide chain) **n= 12**- ^{1}H NMR (400 MHz, $CHCl_3$, δ in ppm): (b,c) 2.07–2.70 (4H, CH2–CH2–C=O benzyl ester), (DMA) 2.88-3.15 (6H, (($CH_3)_2$), (a) 3.83-4.53 (1H, (C=O)-C-H-N),(d) 4.92-5.17 (2H, (C=O)-O-CH2 benzene), (e) 6.45-7.58 (5H, =C-H aromatic), (i) 8.18-8.56 (1H, –NH– of the main polypeptide chain) **n= 14**- ^{1}H NMR (400 MHz, $CHCl_3$, δ in ppm): (b,c) 2.07–2.66 (4H, CH2–CH2–C=O benzyl ester), (DMA) 2.86-3.13 (6H, (($CH_3)_2$), (a) 3.82-4.48 (1H, (C=O)-C-H-N),(d) 4.95-5.19 (2H, (C=O)-O-CH2 benzene), (e) 6.55-7.46 (5H, =C-H aromatic), (i) 8.17-8.49 (1H, –NH– of the main polypeptide chain) **n= 16**- ^{1}H NMR (400 MHz, $CHCl_3$, δ in ppm): (b,c) 2.14–2.63 (4H, CH2–CH2–C=O benzyl ester), (DMA) 2.80-3.13 (6H, (($CH_3)_2$), (a) 3.85-4.32 (1H, (C=O)-C-H-N), (d) 5.06 (2H, (C=O)-O-CH2 benzene), (e) 7.13-7.46 (5H, =C-H aromatic), (i) 8.04-8.62 (1H, –NH– of the main polypeptide chain) **n= 18**- ^{1}H NMR (400 MHz, $CHCl_3$, δ in ppm): (b,c) 2.12–2.63 (4H, CH2–CH2–C=O benzyl ester), (DMA) 2.87-3.11 (6H, (($CH_3)_2$), (a) 3.87-4.17 (1H, (C=O)-C-H-N), (d) 5.05 (2H, (C=O)-O-CH2 benzene), (e) 7.08-7.48 (5H, =C-H aromatic), (i) 8.19-8.51 (1H, –NH– of the main polypeptide chain) **n= 20**- ^{1}H NMR (400 MHz, $CHCl_3$, δ in ppm): (b,c) 2.16–2.65 (4H, CH2–CH2–C=O benzyl ester), (DMA) 2.85-3.08 (6H, (($CH_3)_2$), (a) 3.92-4.35 (1H, (C=O)-C-H-N), (d) 5.06 (2H, (C=O)-O-CH2 benzene), (e) 7.12-7.45 (5H, =C-H aromatic), (i) 8.22-8.50 (1H, –NH– of the main polypeptide chain) **n= 40**- ^{1}H NMR (400 MHz, $CHCl_3$, δ in ppm): (b,c) 2.16–2.61 (4H, CH2–CH2–C=O benzyl ester), (DMA) 2.86-3.04 (6H, (($CH_3)_2$), (a) 3.95 (1H, (C=O)-C-H-N), (d) 5.06 (2H, (C=O)-O-CH2 benzene), (e) 7.13-7.39 (5H, =C-H aromatic), (i) 8.16-8.52 (1H, –NH– of the main polypeptide chain) **n= 60**- ^{1}H NMR (400 MHz, $CHCl_3$, δ in ppm): (b,c) 2.16–2.65 (4H, CH2–CH2–C=O benzyl ester), (DMA) 2.84-3.07 (6H, (($CH_3)_2$), (a) 3.96 (1H, (C=O)-C-H-N),(d) 5.06 (2H, (C=O)-O-CH2 benzene), (e) 7.08-7.47 (5H, =C-H aromatic), (i) 8.17-8.52 (1H, –NH– of the main polypeptide chain) **n= 80**- ^{1}H NMR (400 MHz, $CHCl_3$, δ in ppm): (b,c) 2.16–2.68 (4H, CH2–CH2–C=O benzyl ester), (DMA) 2.80-3.08 (6H, (($CH_3)_2$), (a) 3.96 (1H, (C=O)-C-H-N), (d) 5.06 (2H, (C=O)-O-CH2 benzene), (e) 7.08-7.46 (5H, =C-H aromatic), (i) 8.15-8.55 (1H, –NH– of the main polypeptide chain) **n= 100**- ^{1}H NMR (400 MHz, $CHCl_3$, δ in ppm): (b,c) 2.15–2.60 (4H, CH2–CH2–C=O benzyl ester), (DMA) 2.84-3.11 (6H, (($CH_3)_2$), (a) 3.96 (1H, (C=O)-C-H-N), (d) 5.06 (2H, (C=O)-O-CH2 benzene), (e) 7.08-7.47 (5H, =C-H aromatic), (i) 8.17-8.56 (1H, –NH– of the main polypeptide chain).

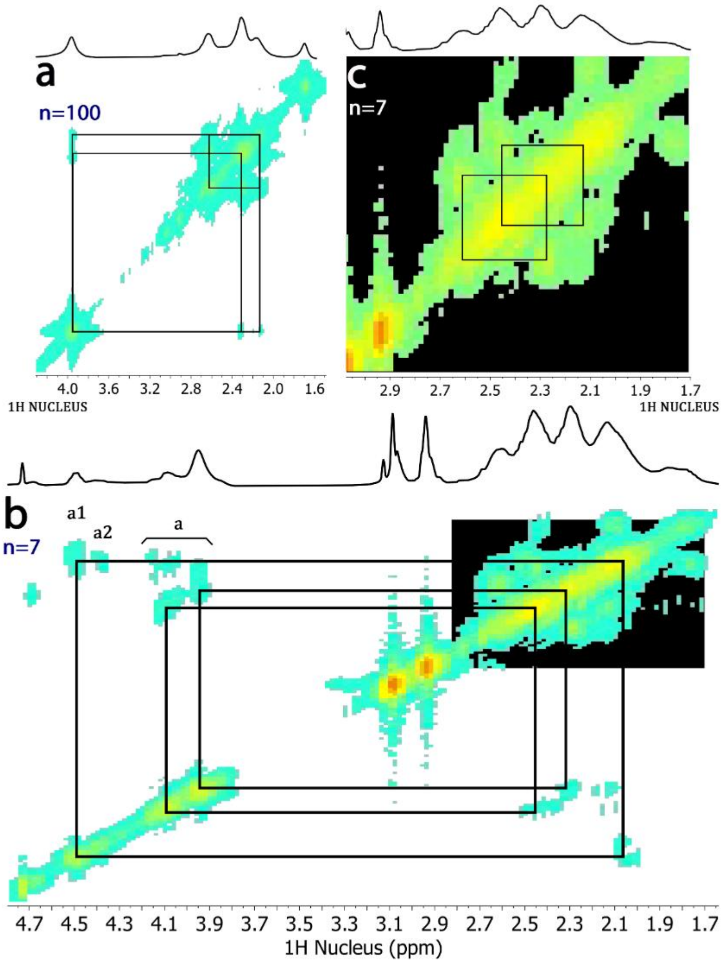

Figure S5. 2D COSY NMR spectra: (a) of 100-monomeric unit homopolymer, (b) 7-monomeric unit homopolymer, (c) 7-monomeric unit homopolymer zoom at lower ppm values.

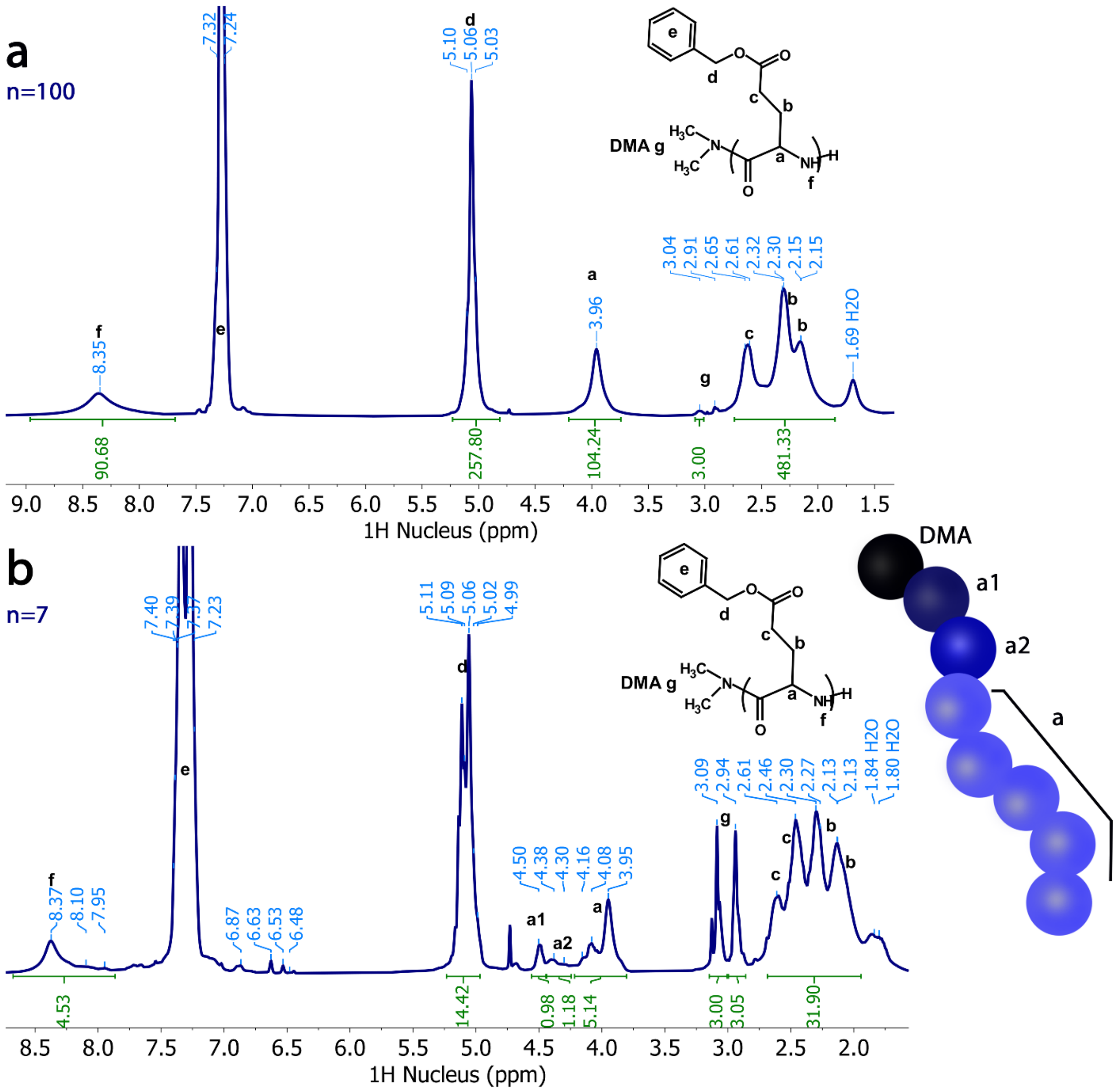

Figure S6. NMR spectra: (a) of 100-monomeric unit homopolymer, (b) 7-monomeric unit homopolymer

C. Size Exclusion Chromatography.

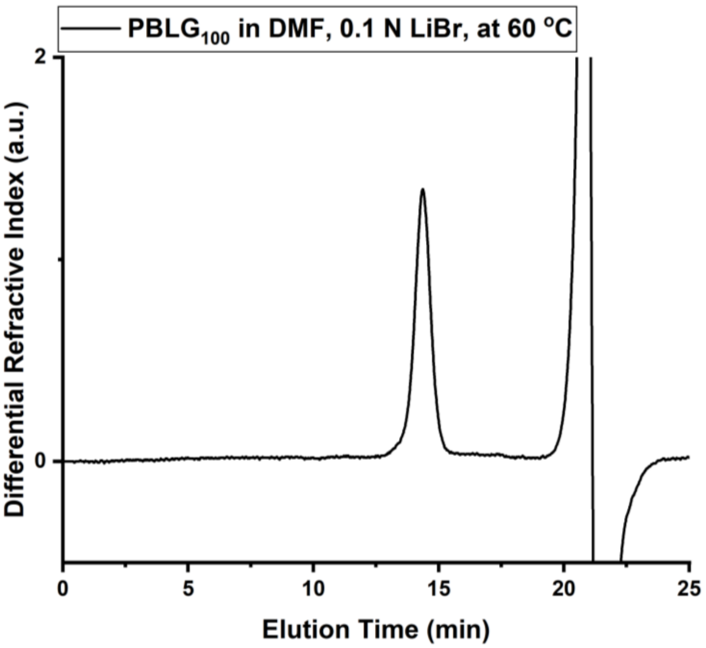

Figure S7. SEC eluogram of $PBLG_{100}$ in DMF, 0.1 LiBr, at 60 ºC,

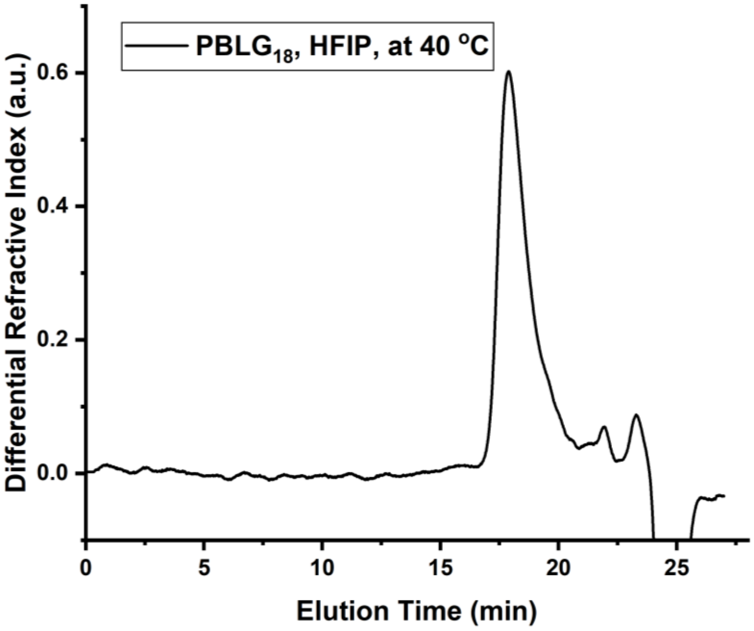

Figure S8. SEC eluogram of $PBLG_{18}$ in HFIP, 0.05% potassium trifluoroacetate at 40 ºC.

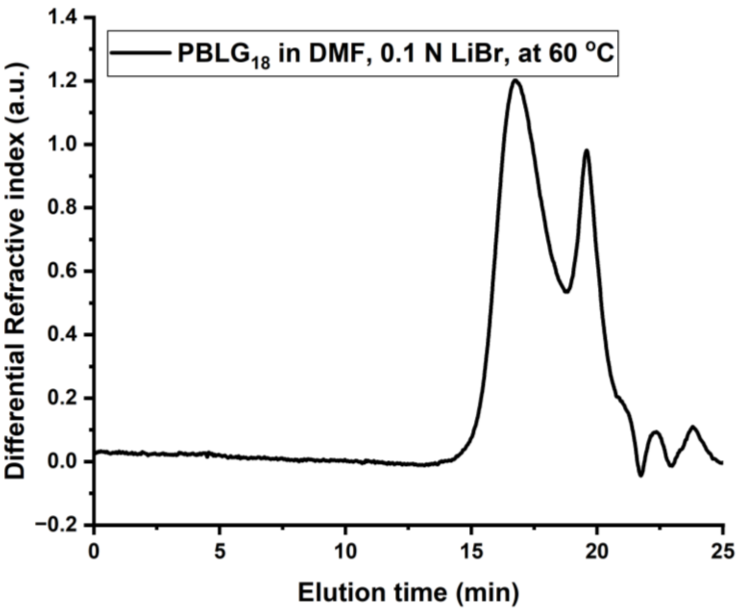

Figure S9. SEC eluogram of PBLG18 in DMF, 0.1 LiBr, at 60 ºC,

II. PHYSICAL PROPERTIES

a. Thermal properties

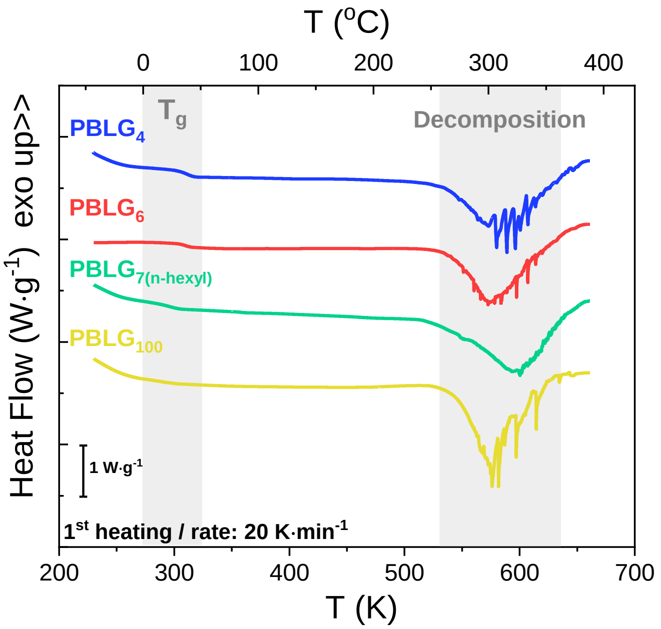

Figure S10. DSC traces of $PBLG_4$ (blue), $PBLG_6$ (red), $PBLG_{7(n\text{-}hexyl)}$ (green) and $PBLG_{100}$ (yellow), obtained during first heating at a rate of 20 K·min^{-1}. Gray areas indicate the respective glass region (low *T*) and the thermal degradation (high *T*). Curves are shifted for clarity.

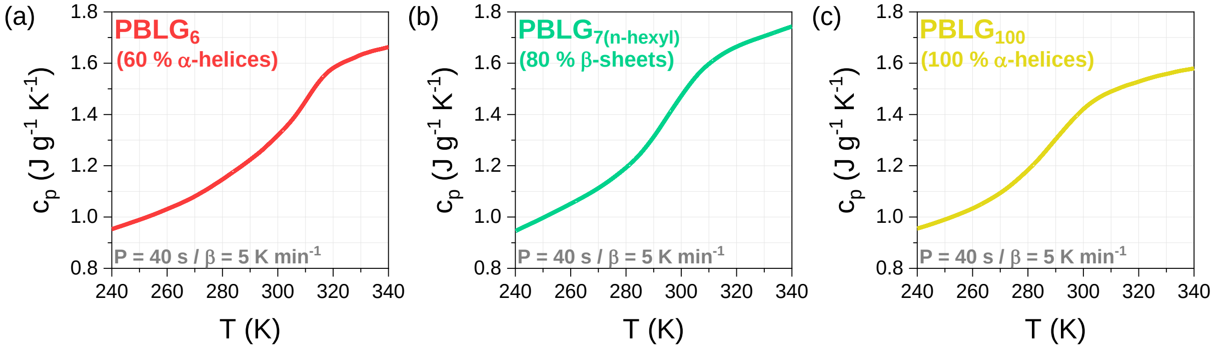

Figure S11. Temperature dependence of the specific (reversing) heat (absolute values) of $PBLG_6$ (red), $PBLG_{7(n\text{-}hexyl)}$ (green) and $PBLG_{100}$ (yellow), obtained from TM-DSC at a period of modulation of P = 40 s (β = 5 K·s^{-1}).

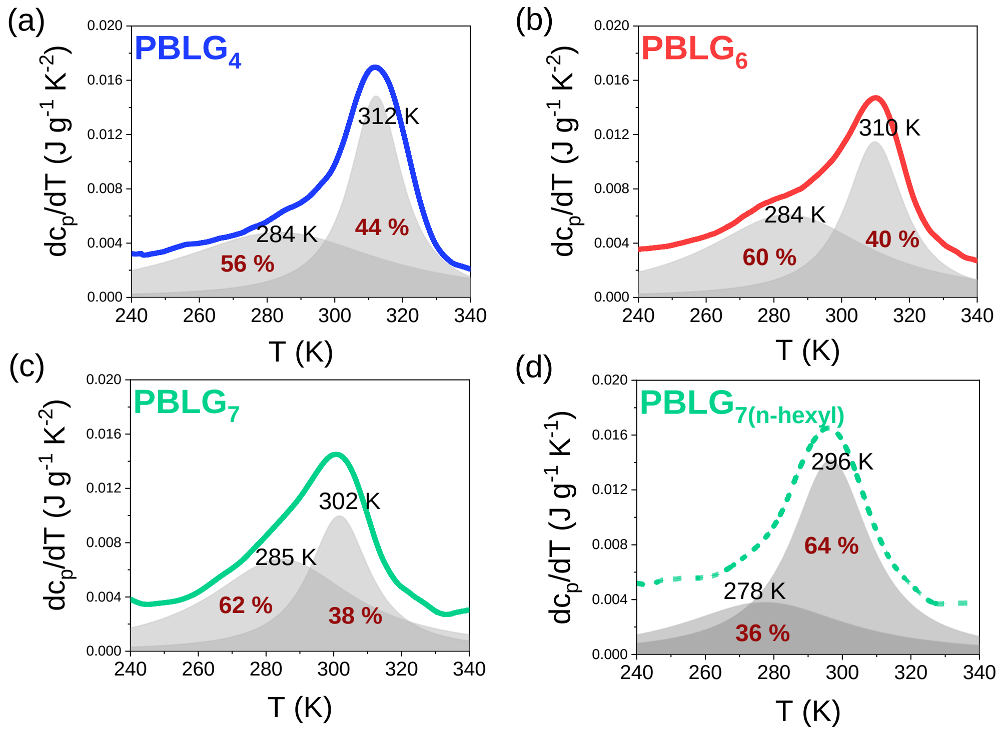

Figure S12. Derivative of the specific heat (obtained from TM-DSC at a period of modulation of P = 40 s) with respect to temperature plotted as a function of temperature for (a) $PBLG_4$, (b) $PBLG_6$, (c) $PBLG_7$ and (d) $PBLG_{7(n\text{-hexyl})}$. Gray integrated areas under each Lorentzian peak were used to estimate the relative $f_{\alpha-\text{helix}}^{\text{DSC}}$ and $f_{\beta-\text{sheet}}^{\text{DSC}}$ of the oligopeptides.

b. Wide-angle X-ray scattering

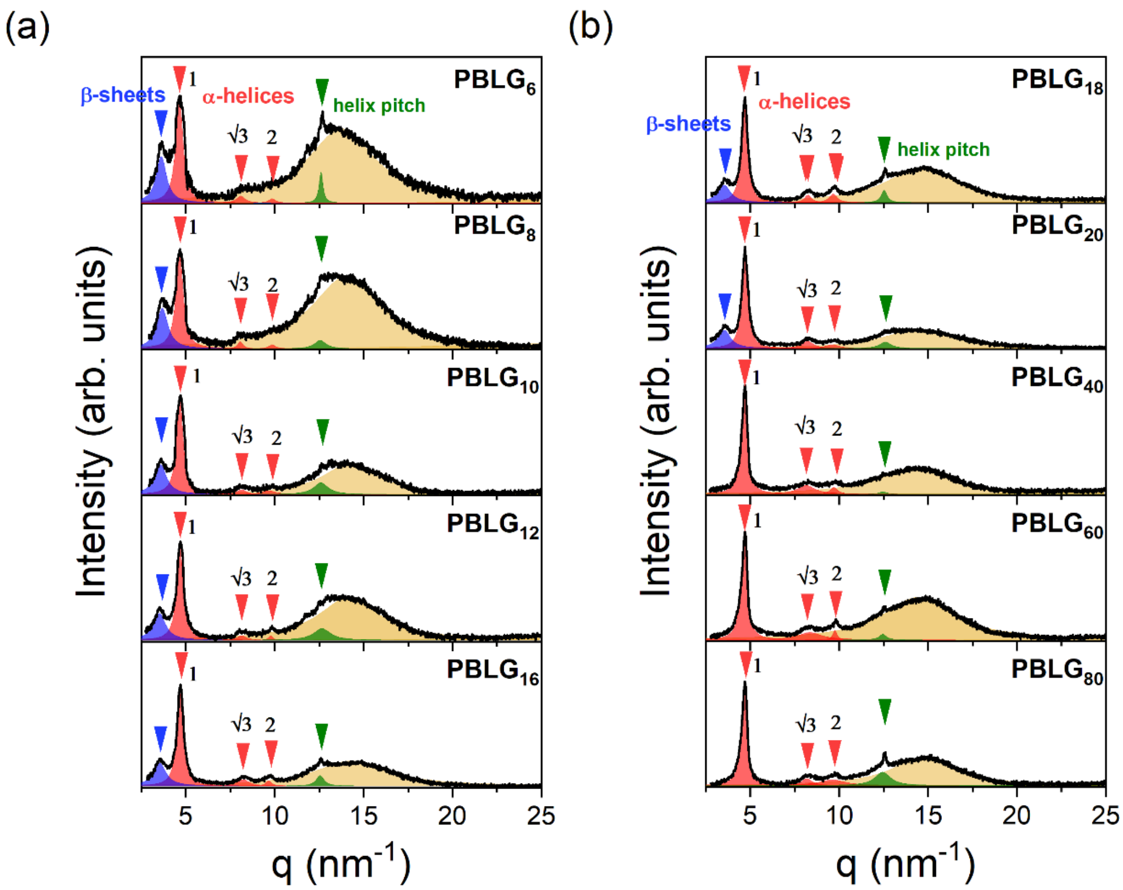

Figure S13. WAXS patterns of the investigated polydisperse samples. Left: $PBLG_{10}$, $PBLG_{12}$, $PBLG_{16}$, $PBLG_{18}$. Right: $PBLG_{20}$, $PBLG_{40}$, $PBLG_{60}$, $PBLG_{80}$. Blue arrows correspond to the lamellar spacing of β-sheet secondary structure. Red arrows indicate the Bragg reflections of the hexagonally packed α-helices, while green arrows give the reflection corresponding to the pitch of the α-helix. The amorphous halo is indicated in yellow.

c. Solid state NMR

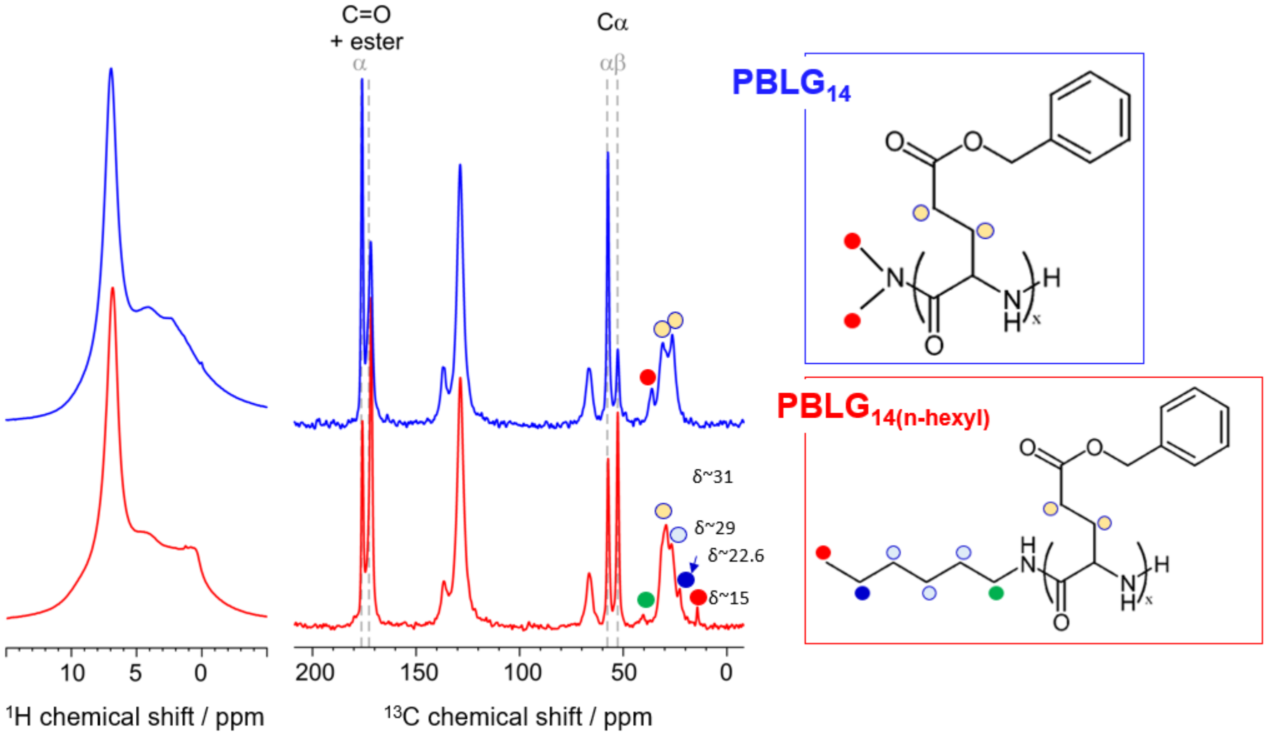

Figure S14. (Center) ^{13}C solid state NMR traces and (left) ^{1}H NMR traces of PBLG$_{14}$ and PBLG$_{14(n\text{-}hexyl)}$ at T = 320 K. The molecular structures of the repeat units of the dimethylamino (up) and n-hexyl (down) terminated polypeptides are also shown, (right) including the color scheme employed for assignment purposes.

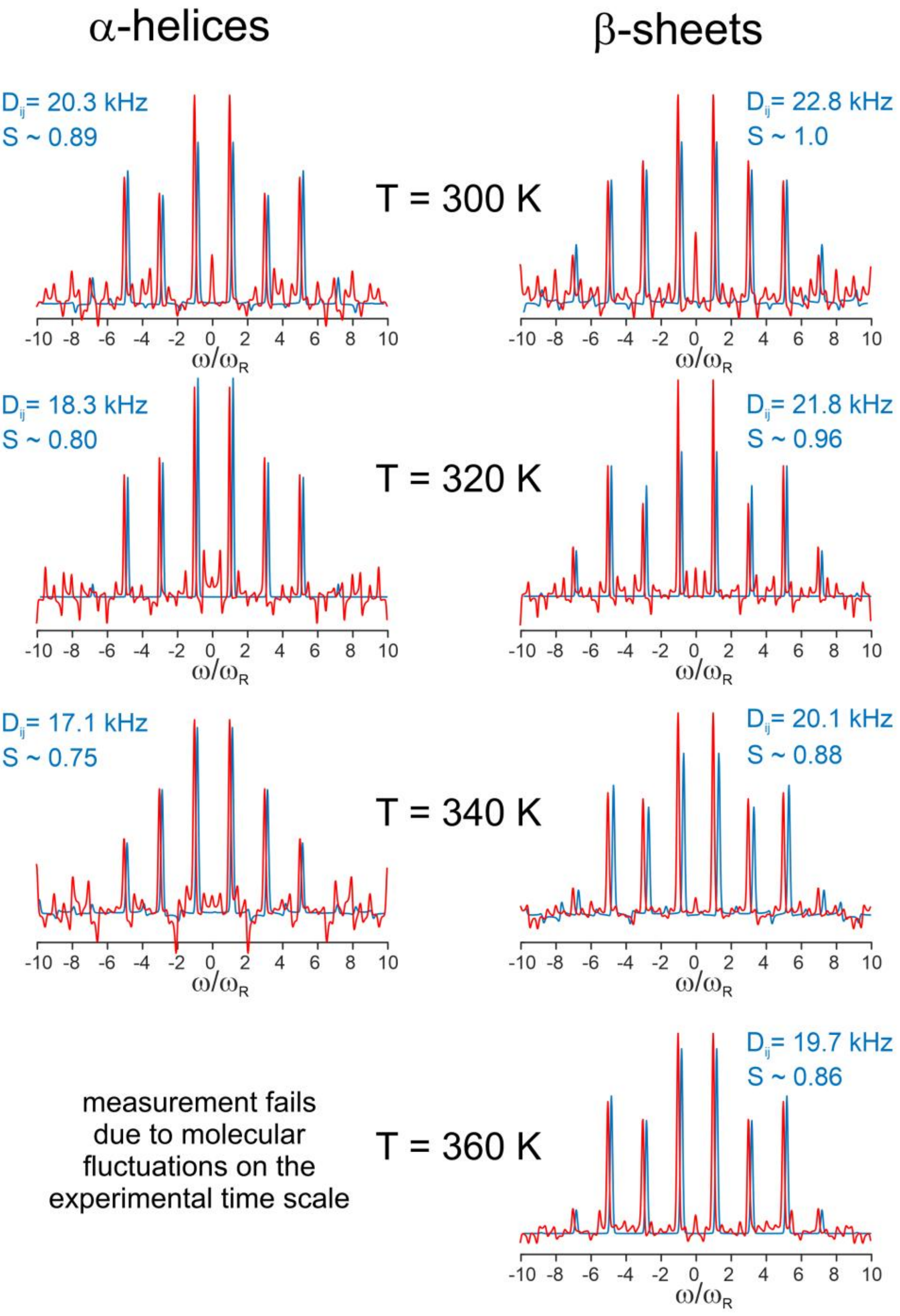

Figure S15. ^{13}C-^{1}H REREDOR spinning sideband patterns recorded at 25 kHz spinning at the magic angle for $PBLG_{14}$ shown for different temperatures.

d. Dielectric Spectroscopy (DS)

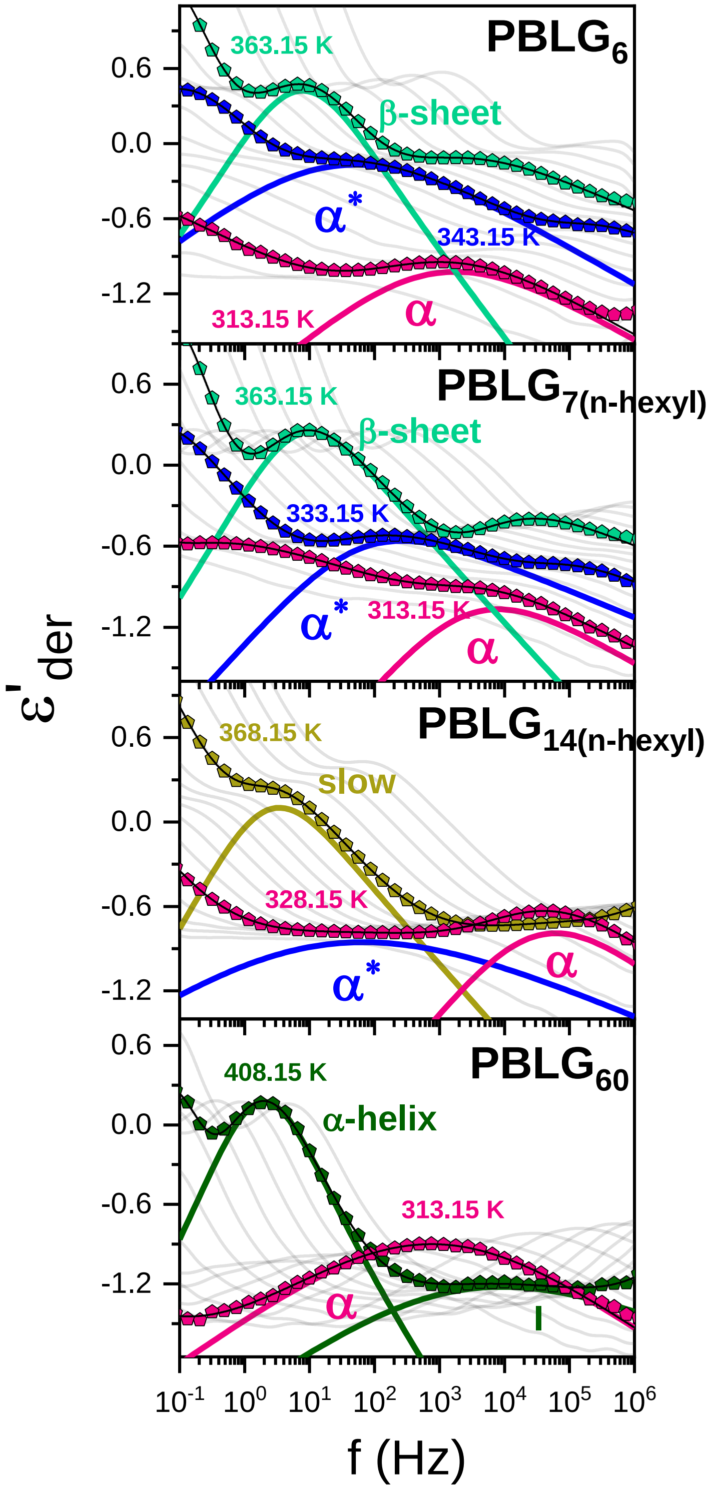

Figure S16. Derivative of dielectric permittivity as a function of frequency at selected temperatures, for four distinct samples $PBLG_6$, $PBLG_{7(n\text{-}hexyl)}$, $PBLG_{14(n\text{-}hexyl)}$ and $PBLG_{60}$. Some temperatures are highlighted for each sample showing simulations of the HN function for the respective processes: α segmental process (pink), α^* segmental process (blue), β-sheet process (green), slow process (yellow), α-helix process (dark green).

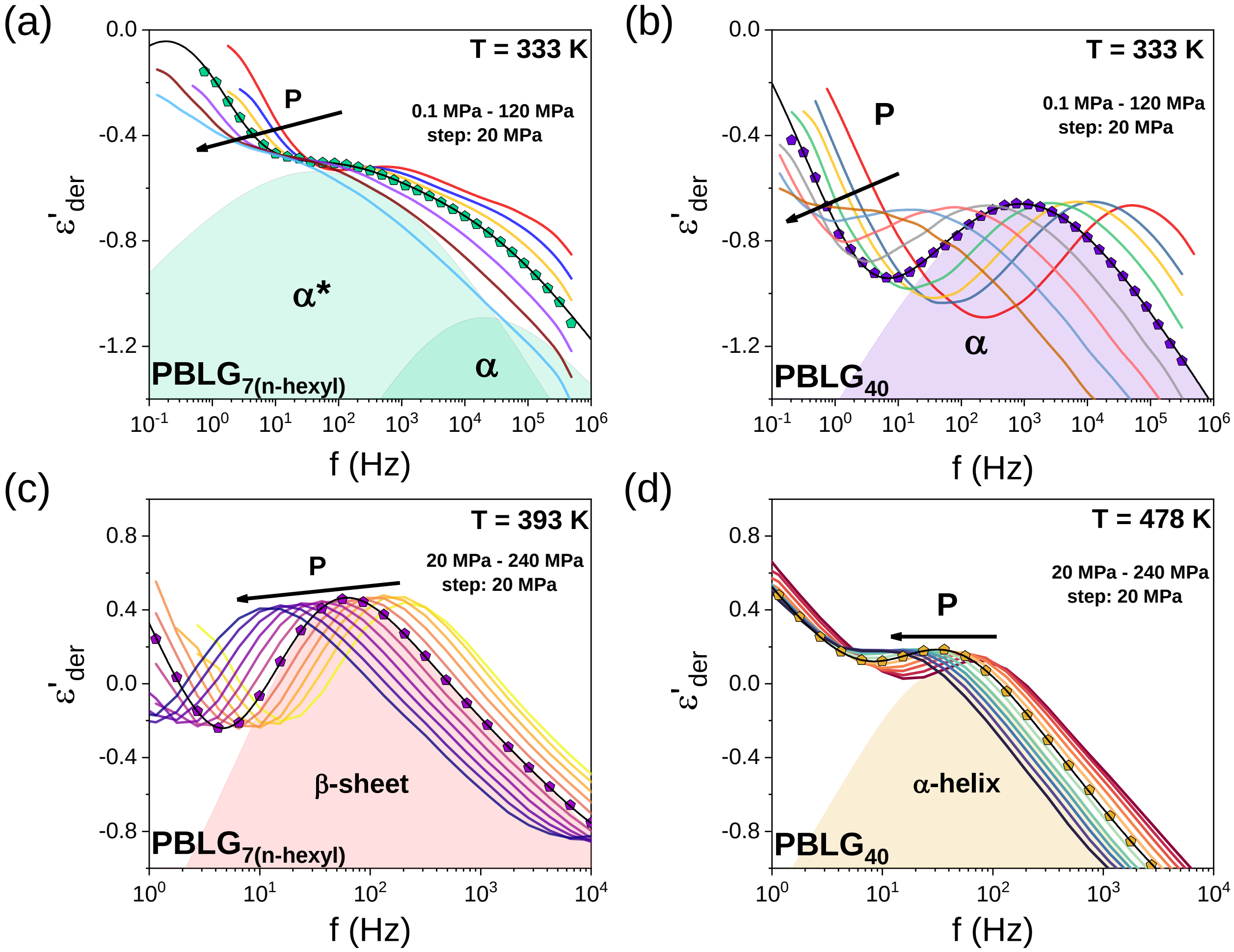

Figure S17. Derivative of dielectric permittivity as a function of frequency under isothermal conditions at (a) 333 K and (c) 393 K for $PBLG_{7(n\text{-}hexyl)}$ and at (b) 333 K and (d) 478 K for $PBLG_{40}$. Pressure increases in the direction of the arrow. The colored areas for the highlighted pressures (indicated with symbols) represent fits (simulations) to eq (5) for the respective processes.

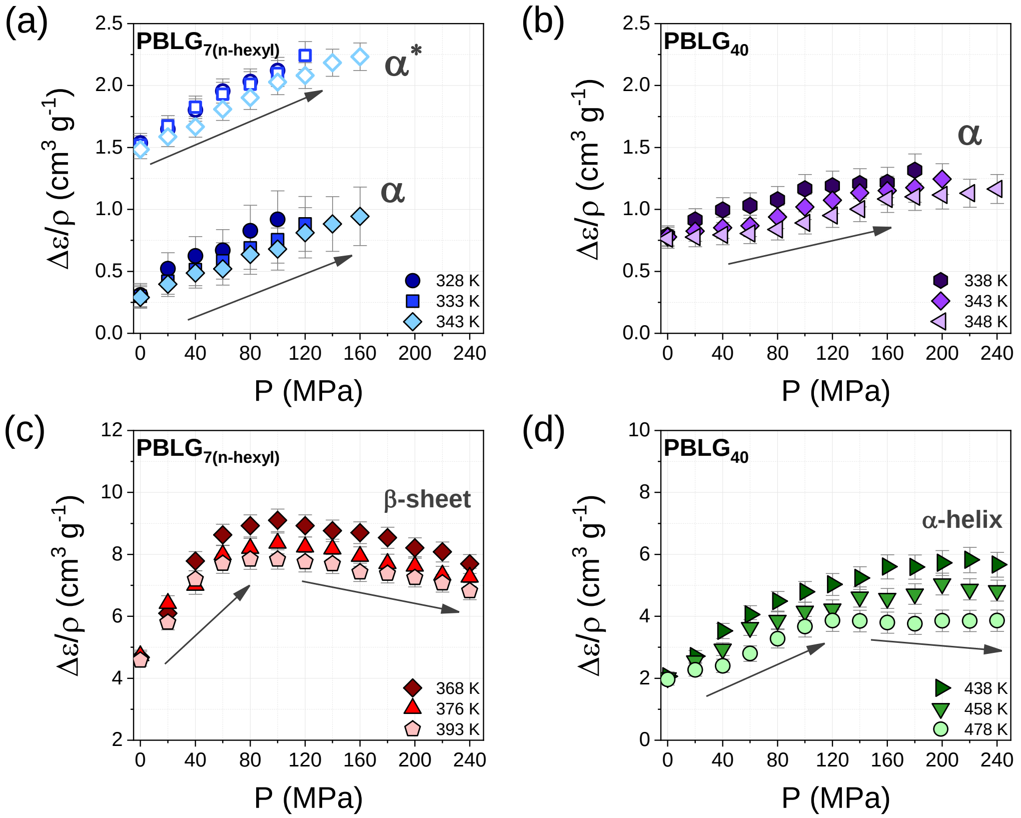

Figure S18. Normalized dielectric strengths, $\Delta\varepsilon/\rho$ for $PBLG_{7(m)}$ corresponding to (a) the α and α^* process and (c) the β-sheet process, and for $PBLG_{40}$ indicating (b) the α process and (d) the α-helix process at some selected temperatures.

e. Viscoelasticity

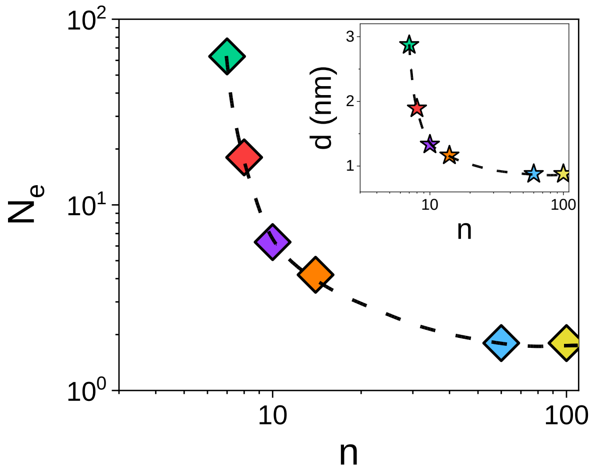

Figure S19. Dependence of the characteristic length N_e between the structural constraints (see text), as a function of molar mass. Different colors indicate the different peptides: $PBLG_7$ (green), $PBLG_8$ (red), $PBLG_{10}$ (purple), $PBLG_{14}$ (orange), $PBLG_{60}$ (blue) and $PBLG_{100}$ (yellow). The inset shows the characteristic length *d* between the structural constraints as a function of *n*. The dashed lines are guides for the eye.

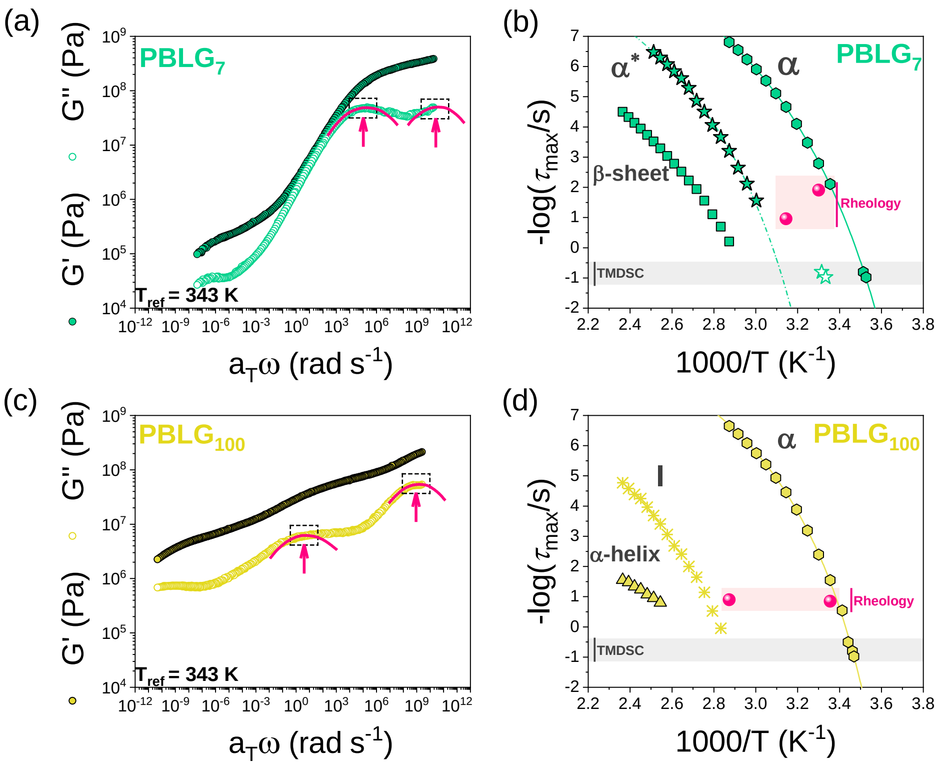

Figure S20. Master curves of the storage (filled circles) and loss (open circles) moduli of (a) $PBLG_7$ (green) and (c) $PBLG_{100}$ (yellow). The insets show the frequency of the maximum of G'' at the respective temperatures. The characteristic times obtained as $\tau = 1/\omega$ are indicated in the Arrhenius plots of (b) $PBLG_7$ and (d) $PBLG_{100}$.

III. REFERENCES

(1) Mavrogiorgis, D.; Bilalis, P.; Karatzas, A.; Skoulas, D.; Fotinogiannopoulou, G.; Iatrou, H. Controlled polymerization of histidine and synthesis of well-defined stimuli responsive polymers. Elucidation of the structure–aggregation relationship of this highly multifunctional material. *Polymer Chemistry* **2014**, *5* (21), 6256-6278, 10.1039/C4PY00687A. DOI: 10.1039/C4PY00687A.
